# Design, characterization, DFT studies, and molecular docking of new benzofuran–pyrazol-acrylamide hybrids as insecticidal agents against *Spodoptera littoralis* and *Tribolium castaneum*

**DOI:** 10.1038/s41598-026-39839-z

**Published:** 2026-03-25

**Authors:** Ghada G. El-Bana, Mohamed R. Fouad, Ahmed D. H. Deeb, Ahmed M. Wahba, Ahmed F. El-Sayed, Ghada E. Abd el-Ghani

**Affiliations:** 1 Department of Chemistry, Faculty of Science, Mansoura University, El-Gomhoria Street, Mansoura, 35516 Egypt; 2 Mansoura University Student’s Hospital, Mansoura University, El-Gomhoria Street, Mansoura, 35516 Egypt; 3 Department of Pesticide Chemistry and Technology, Faculty of Agriculture, Alexandria University, Aflaton St., El-Shatby, Alexandria, 21545 Egypt; 4 Department of Physical Sciences, Chemistry Division, College of Science, Jazan University, 45142 Jazan, Saudi Arabia; 5 Medical Sciences and Preparatory Year Department, Northern Border Province, North Private College of Nursing, 73312 Arar, Kingdom of Saudi Arabia; 6 Basic Science Department, Higher Institute of Engineering and Technology (HIET), El-Mahala El-Kobra, 31951 Egypt; 7 Microbial Genetics Department, Biotechnology Research Institute, National Research Centre, Giza, Egypt; 8 Microbial Genetics, Egypt Center for Research and Regenerative Medicine (ECRRM), Cairo, Egypt

**Keywords:** Benzofuran, Pyrazole, Insecticide bioassay DFT studies, Molecular docking, ADMET, Dynamics (MD) simulations, Chemical biology, Chemistry

## Abstract

This study discusses the synthesis and structural characterization of a new series of benzofuran-pyrazole cyanoacrylamide derivatives **3a–f** and **5** utilizing spectroscopic analyses involving IR, NMR, and ESI-MS spectrometry. The insecticidal activity of benzofuran-pyrazole cyanoacrylamide derivatives **3a–f** and **5** against *Spodoptera littoralis* and *Tribolium castaneum* was evaluated. The benzofuran-pyrazole cyanoacrylamide derivatives on *Spodoptera littoralis* and *Tribolium castaneum* revealed that **3b**, **3c**, and **3a** were the most effective compounds, respectively, while compounds **3d**,** 3e**,** 3f**, and **5** were not lethal to *S. littoralis*. The LC_50_ values for compounds **3b**, **3c**, and **3a** were 79.58, 106.37, and 226.17 ppm at 72 h, respectively; while in contrast, compounds **1**, **3d**,** 3e**,** 3f**, and **5** exhibited very low or no contact toxicity against *T. castaneum*. Density Functional Theory (DFT) calculations were employed to compare the E/Z stereoisomers. The E isomers of **3a**, **3c**, **3d**, and **3e** exhibited lower energy gaps, higher softness, and greater electrophilicity, with **3c.**E emerging as the most reactive candidate. In contrast, compounds **3b**, **3f**, and **5** favored the Z configuration, showing improved orbital overlap and higher dipole moments. These findings confirm that stereochemistry strongly influences electronic structure, reactivity, and potential adsorption behavior. In addition, a computational evaluation was conducted to assess the potential of novel benzofuran-indazole hybrid compounds (**3a**, **3b**, **3c**) as insecticidal agents targeting acetylcholinesterase (AChE) from two major pests: *Spodoptera litura* (tobacco cutworm) and *Tribolium castaneum* (red flour beetle). Molecular docking revealed that all three compounds exhibited strong binding affinities (ranging from − 8.0 to − 8.8 kcal/mol) to both insect AChEs, comparable to or surpassing the positive control (carbaryl) and co-crystallized inhibitor (NAF). The binding modes involved key interactions with catalytic and peripheral anionic site residues, facilitated by hydrogen and hydrophobic bonds. Compound **3b** consistently showed among the highest affinities. Subsequent molecular dynamics simulations of the **3b**-AChE complexes confirmed stable binding, with minimal perturbation to protein backbone stability, maintained compactness, and persistent intermolecular hydrogen bonds. ADMET predictions indicated that the compounds generally comply with drug-likeness rules. Toxicity risks (mutagenic, tumorigenic) were predicted to be low. Overall, the in silico analyses identify these hybrid compounds, particularly **3b**, as promising, stable, and specific AChE inhibitor leads for the development of novel insecticides.

**Supplementary Information:**

The online version contains supplementary material available at 10.1038/s41598-026-39839-z.

## Introduction

**Fig. 1:**
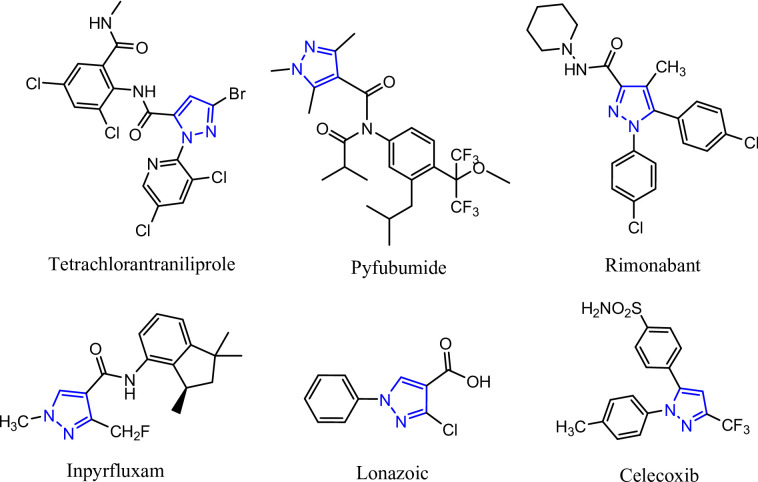
Chemical structures of representative amide insecticides and fungicides.

Heterocyclic ring systems and a large range of naturally occurring bioactive chemicals share structural commonalities that result in robust backbones with a multitude of biological characteristics^1^. For instance, benzofuran is a basic structural component of many biologically active natural and synthetic products, including analgesics, anti-inflammatory, anti-hyperglycemic, anticancer, antiviral, antibacterial, antifungal, anti-Alzheimer’s disease, and insecticides^2–10^. Synthetic strategies for production derivatives of the pyrazole ring structure are necessary to strengthen the modern organic synthesis toolkit, and this structure has also been widely observed as a pharmacophore. The presence of pyrazoles in naturally occurring chemicals and their diverse biological actions have captivated thousands of researchers worldwide^11^. Furthermore, pyrazoles, as heterocycles containing nitrogen, exhibit biological activities similar to those of several pesticide compounds in medicinal chemistry, including insecticides and fungicides^12^ (Fig. 1). Recently, the pyrazole nucleus and benzofuran ring hybridization have been recognized as a medically promising agent, acting as a vital scaffold in the development and synthesis of compounds with promising antibacterial, antioxidant, and anti-inflammatory characteristics^13–15^. Also, it has been demonstrated that the acrylamide moiety is a useful tool for creating new medicinal entities. The Acrylamide derivatives exhibit a wide range of biological activities, such as antitumor activity, antiviral activity, anti-inflammatory activity, antidiabetic activity, tubulin polymerization inhibitor activity, antibacterial activity, antifungal activity, and angiotensin II receptor antagonist activity^16–20^. The compounds that contained acrylamides, thioxoacetamide, and hydrazinyl had good action against Spodoptera littoralis larvae and had a binding affinity to the target enzyme^21^. Additionally, the pyrazole derivatives exhibited aphicidal properties against the corn leaf aphid (*Rhopalosiphum maidis*) and the mealy plum aphid (*Hyalopterus pruni*)^22^. *Spodoptera littoralis*, also referred to as the Egyptian cotton leafworm or African cotton leafworm, is a species of moth in the family Noctuidae. *S. littoralis* is widely distributed on most continents of the world, especially Africa, Asia, and Europe^23^. This moth is a polyphagous pest of various ornamental plants and horticultural crops across the globe, infesting dozens of cultivated plants belonging to 44 families, including cotton, alfalfa, soybeans, sweet potato, pepper, tomato, eggplant, lettuce, maize, strawberry, and wheat^24^.

*S. littoralis* causes yield losses of up to 50%, typically due to larval foraging activity^25^. It feeds on young leaves, stems, buds, shoots, almonds, and fruits^26^. As a result, this species was assigned the label of A2 quarantine pest by the European and Mediterranean Plant Protection Organization and was cautioned as a highly invasive species in the United States^27^. Its infestation can also produce the subsequent development of pathogens on damaged plant tissues and can lead to significant product losses^28^. The devastating impacts caused by this pest have led to the development of both chemical and biological control methods^29,30^.

Losses of cereal grains in storage can reach up to 20% of overall production, and stored product insect pests are a primary factor in these losses. The red flour beetle, *Tribolium castaneum* (Coleoptera: Tenebrionidae), is among the most destructive and universal pests found in grains and processed products, and also considered as the representative pest in flour mills^31,32^. Globally, *T. castaneum* is widely distributed in tropical and warm regions, mainly in Africa, South Asia and South America. It causes both qualitative and quantitative loss of produce in storage areas that are not properly secured and are exposed to the outside or when there is insufficient storage space and produce is left in the open^33^. This pest species is an effective model for developmental, physiological, and applied insect research on coleopteran species. In addition to direct losses, this beetle secretes benzoquinones, which are carcinogenic substances with an unpleasant and lingering odor that alter the flour’s color and pose serious health concerns to humans. Therefore, it is essential to create more targeted, and environmentally friendly control approaches to address widespread insect pests^34^. There are many commercial pesticides against pests in the Egyptian pesticide market, but only a few commercial pesticides are available against *S. littoralis* and *T. castaneum*. Additionally, the emergence of resistance in these pests to pesticides used in the control process has occurred. Thus, this study aimed to synthesize eight benzofuran-based pyrazole nucleus derivatives and determine their toxicity to these pests, in an attempt to find new pesticides that are more effective and safer for the environment and humans. Also, DFT calculations were performed in order to gain a deeper understanding of the structure-reactivity relationships of the synthesized compounds^35^. Based on the calculated global reactivity descriptors, the experimentally observed differences in reactivity and biological activity could be explained at the molecular level. Furthermore, DFT analysis explained how stereochemistry influenced the electronic structure of the molecules and their interactions with biological targets. Furthermore, molecular docking was used in this study to investigate the interactions between newly synthesized compounds and specific protein receptors, thereby assessing their proposed mechanisms of action and anti-cancer efficacy, as demonstrated in previous studies^36^.

Thus, the purpose of this work was to synthesize an acrylamides derivatives block with a pyrazole core in order to study its insecticidal action against *Spodoptera littoralis* larvae and *Tribolium castaneum* as well as its activity towards nucleophilic reagents and the detection of resistance levels in the field strain.

## Results and discussion

### Chemistry

**Scheme 1:**
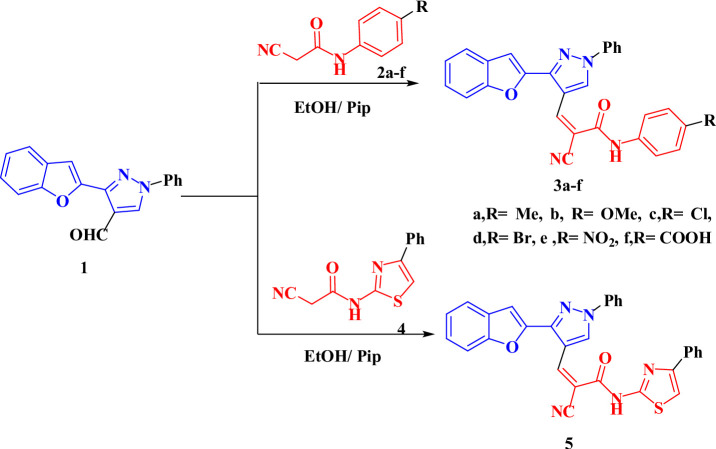
Synthesis of benzofuran-pyrazole cyanoacrylamide derivatives **3a–f**, and **5**.

The Knoevenagel condensation (KC) is a multifaceted reaction which involves carbonyl groups and various “active methylenes,” yielding a broad variety of small molecules with significant utility across diverse fields of chemistry, encompassing synthetic organic, organic solar cells, polymer chemistry, medicinal, pharmaceutical, and cosmetics^37,38^. Our investigation began by using 3-(benzofuran-2-yl)-1-phenyl-1*H*-pyrazole-4-carbaldehyde (**1**) as the carbonyl compound to react with cyanoacetamide derivatives **2a–f** and **4** in refluxing EtOH (10 ml) with piperidine for 30 min to yield the desired products **3a–f** and **5** in good yields (Scheme 1). The spectrum investigations of the new benzofuran-pyrazole cyanoacrylamide derivatives **3a–f** and **5** validated their chemical structures. The IR spectra for compounds **3a–e** indicated the presence of hydroxyl and nitrile groups, exhibiting vibrations at 3274–3333 cm^− 1^ (NH group), 2211–2217 cm^− 1^ (CN group), 1614–1676 cm^− 1^ (C=O), and 1592–1598 cm^− 1^ (C=N). The ^1^H-NMR spectra of **3a** and **3b** exhibited new singlet signals, each integrating for three protons, corresponding to methyl and methoxy protons at *δ* = 2.30 and 3.77, respectively. Additionally, three singlet signals (one proton each) were observed at *δ* = 7.55 and 7.56 for CH_furan_, 8.59 for CH = C, 9.25 and 9.26 for CH_pyrazole_, and 10.31 and 10.37 for NH protons, respectively. The ^13^C-NMR spectra of compounds **3a** and **3b** each exhibited 24 carbon signals, with distinctive carbons at *δ* = 21.00 ppm (methyl, **3a**) and 55.70 ppm (methoxy, **3b**). Additionally, the mass spectra of **3a** and **3b** exhibited molecular ion peaks at *m/z* = 444.79 (*M*^*+*^, 43.29%) and 460.40 (*M*^*+*^, 29.46%), respectively.

Once more, the ^1^H-NMR spectra of compounds **3c–e** exhibited three singlet signals (one proton each) at *δ* = 7.57 for CH_furan_, 8.61–8.66 for CH = C, 9.25–9.27 for CH_pyrazole_, and 10.56–10.96 for NH protons. The ^13^C-NMR spectra revealed characteristic signals at *δ* = 106.00–106.45, 115.27–117.01, 148.15–148.23, and 160.72–161.40 ppm for CH_furan_, CN, =CH, and C=O, respectively. Furthermore, the mass spectrum of compound **3c–e** exhibited *m/z* values of 460.40 (*M*^*+*^, 29.46%), 508.41/510.48 (*M*^*+*,^ *M*^*+*^+2, 12.73/ 15.64%), and 475.74 (*M*^*+*^, 9.55%), respectively.

Additionally, the ^1^H-NMR spectrum of hydrazono derivative compound **3f** exhibited singlet signals corresponding to CH_furan_, CH=C, CH_pyrazole_, NH, and OH protons at *δ* = 7.57, 8.62, 9.27, and 10.56 ppm, respectively. The ^13^C-NMR spectrum of compound **3f** exhibited 23 carbon signals, with distinctive signals at *δ* = 106.45, 116.70, 148.23, and 160.73 ppm corresponding to CH_furan_, CN, =CH, and C=O, respectively. Additionally, the mass spectrum of compound **3f** exhibited an *m/z* of 474.31 (*M*^*+*^, 17.13%).

Thiazole, also known as 1,3-thiazole, is a heterocyclic molecule with five members that combines nitrogen and sulfur. Common pharmacologically active compounds include vitamin B, penicillin-like antibiotics, pramipexole, meloxicam, and nizatidine, all of which contain the thiazole ring^39,40^. Thus, the expected 3-(3-(benzofuran-2-yl)-1-phenyl-1*H*-pyrazol-4-yl)-2-cyano-*N*-(4-phenylthiazol-2-yl)acrylamide (**5**) was formed *via* condensation of starting compound **1** with 2-cyano-*N*-(4-phenylthiazol-2-yl)acetamide (**4**) (Scheme 1). Spectral analyses were used to characterize the structure of compound **5**. Compound **5’s** IR spectrum revealed absorption peaks at *v* = 3403, 2207, 1676, and 1583 cm^–1^, respectively, confirming the existence of OH, CN, C=O, and C=N groups. Also, the ^1^H-NMR spectra of pyrazolo derivative **5** exhibited three new singlet signals at *δ* = 8.75, 9.29, and 13.16 ppm, these corresponds to = CH, CH_pyrazole_, and OH protons, respectively. In addition, the mass spectrum of compound **5** exhibited an ion peak at *m/z* = 513.56 (*M*^*+*^, 30.87%), corresponding to the molecular formula C_30_H_19_N_5_O_2_S, which corresponds with its proposed structure.

### Insecticidal activity against *Spodoptera littoralis* and *Tribolium castaneum*

#### Toxicity of chemicals to *Spodoptera littoralis*

**Fig. 2:**
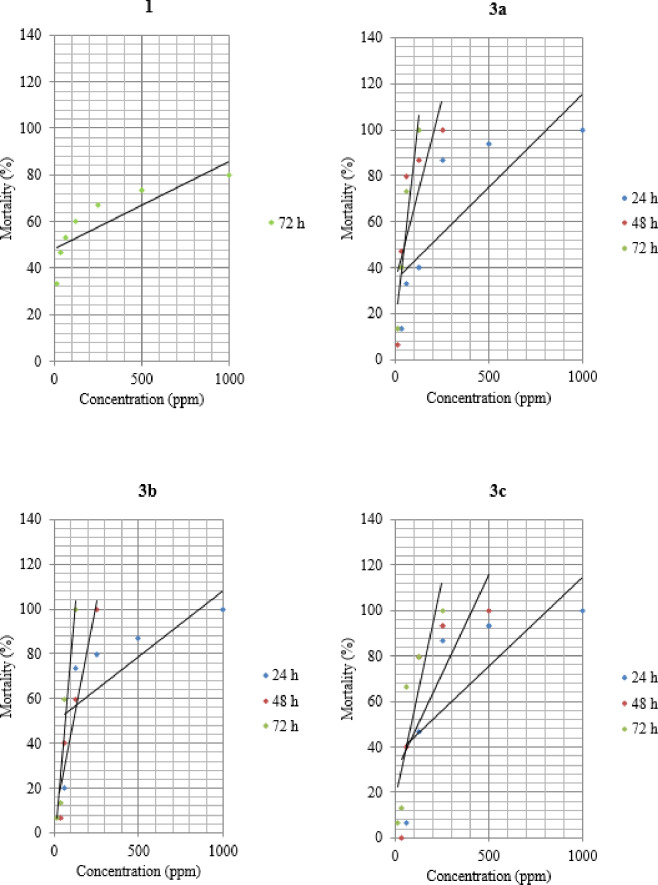
Mortality analysis of *S. littoralis* (4rd instar larvae) to concentratio–response.

The data in Table 1 represented the LC_5_, LC_50_, & LC_95_ values and other parameters of compound **1** at 72 h post-treatment, and compounds **3a**,** 3b** and **3c** at 24, 48, and 72 h post-treatment against the 4th larval instar of *S. littoralis*. The results displayed that the LC_50_ values were 107.77 (77.52–149.64), 38.46 (28.72–51.44), 106.10 (70.44–159.16), 83.53 (64.30–108.51), and 141.24 (108.70–183.44), and 77.10 (59.26–100.25) ppm at 24 and 48 h for **3a**,** 3b** and **3c** respectively, while the LC_50_ values were 52.94 (18.71–142.67), 36.11 (27.62–47.18), 49.57 (39.10–62.85), and 55.47 (42.26–72.81) ppm at 72 h for **1**, **3a**, **3b**, and **3c**, respectively. Compounds **3d**,** 3e**,** 3f**, and **5** were not lethal to the *S. littoralis*. No mortality was noticed in the untreated control group during the entire 72-h observation period of 72 h. However, compound 1 didn’t cause significant mortality, even 48 h after application. The highest mortality (80%) was noted with 1000 ppm 72 h after application (Table 2). Based on LC50 values, the toxicity of benzofuran-pyrazole derivatives to *S. littoralis* is ranked from most to least toxic as follows: **3a** > **3b** > **3c** > **1** (Fig. 2).

The indiscriminate deployment of both traditional and newer insecticides has led to the development of resistance to nearly all insecticides within the Noctuidae family, especially *S. littoralis*. Therefore, we tested eight benzofuran derivatives as potential new insecticides on *S. littoralis*. The findings of this study align with the work of Williams et al. 1994 and Díaz et al. 2023^41,42^, who investigated benzofuran derivatives on S. littoralis.

**Table 1:** The lethal concentrations of tested Benzofuran derivatives against the 4rd instar *S. littoralis* larvae at laboratory conditions.

Compounds	Time (h)	LC_5_ (F.L)	LC_50_ (F.L)	LC_95_ (F.L)	Slope ± SE	*P*	Chi-square
1	72	0.18 (0.01–11.20)	52.94 (18.71–142.67)	16,295.53 (636.89–573452.5)	0.66 ± 0.05	1	0.12
3a	24	21.22 (10.43–42.48)	107.77 (77.52–149.64)	547.48 (297.24–1021.68)	2.33 ± 0.17	0.67	2.39
	48	10.70 (5.72–19.67)	38.46 (28.72–51.44)	138.33 (81.25–238.61)	2.96 ± 0.34	0.71	2.38
	72	11.89 (6.76–20.66)	36.11 (27.62–47.18)	109.63 (65.79–184.71)	3.41 ± 0.53	0.62	0.94
3b	24	19.65 (6.99–54.04)	106.10 (70.44–159.16)	572.98 (290.21–1145.80)	2.25 ± 0.27	0.61	3.87
	48	28.15 (16.62–47.31)	83.53 (64.30–108.51)	247.90 (149.04–415.35)	3.48 ± 0.53	0.31	2.36
	72	19.72 (12.65–30.52)	49.57 (39.10–62.85)	124.63 (79.71–169.18)	4.11 ± 0.73	0.20	3.22
3c	24	48.22 (28.30–81.67)	141.24 (108.70–183.44)	413.65 (257.12–668.99)	3.52 ± 0.52	0.83	1.63
	48	25.73 (15.17–43.29)	77.10 (59.26–100.25)	231.03 (143.24–375.27)	3.45 ± 0.47	0.94	0.57
	72	16.70 (9.84–28.05)	55.47 (42.26–72.81)	184.21 (111.05–308.74)	3.16 ± 0.35	0.69	2.37

LC, Lethal concentrations; F.L, Fiducial limits; SE, Standard error; P, Probability.

**Table 2:** Mortality (%) ± SE of Benzofuran derivatives against the 4rd instar *S. littoralis* larvae at laboratory conditions.

Compounds	Time(h)	Concentrations (ppm)						
		15.62	31.25	62.5	125	250	500	1000
1	72	33.33 ± 0.67	46.67 ± 0.88	53.33 ± 1.45	60.00 ± 0.00	66.67 ± 1.67	73.33 ± 0.88	80.00 ± 0.58
3a	24	0.00 ± 0.00	13.33 ± 0.33	33.33 ± 1.20	40.00 ± 1.15	86.67 ± 0.33	93.33 ± 0.33	100.00 ± 0.00
	48	6.67 ± 0.33	46.67 ± 0.88	80.00 ± 0.00	86.67 ± 0.33	100.00 ± 0.00	100.00 ± 0.00	100.00 ± 0.00
	72	13.33 ± 0.33	40.00 ± 1.00	73.33 ± 0.67	100.00 ± 0.00	100.00 ± 0.00	100.00 ± 0.00	100.00 ± 0.00
3b	24	0.00 ± 0.00	0.00 ± 0.00	20.00 ± 0.58	73.33 ± 0.33	80.00 ± 0.00	86.67 ± 0.33	100.00 ± 0.00
	48	0.00 ± 0.00	6.67 ± 0.33	40.00 ± 0.58	60.00 ± 1.53	100.00 ± 0.00	100.00 ± 0.00	100.00 ± 0.00
	72	6.67 ± 0.33	13.33 ± 0.67	60.00 ± 0.00	100.00 ± 0.00	100.00 ± 0.00	100.00 ± 0.00	100.00 ± 0.00
3c	24	0.00 ± 0.00	0.00 ± 0.00	6.67 ± 0.33	46.67 ± 1.20	86.67 ± 0.33	93.33 ± 0.33	100.00 ± 0.00
	48	0.00 ± 0.00	6.67 ± 0.33	40.00 ± 0.58	80.00 ± 0.00	93.33 ± 0.33	100.00 ± 0.00	100.00 ± 0.00
	72	6.67 ± 0.33	13.33 ± 0.33	66.67 ± 0.67	80.00 ± 1.00	100.00 ± 0.00	100.00 ± 0.00	100.00 ± 0.00

#### Toxicity of chemicals to *Tribolium castaneum*

**Fig. 3:**
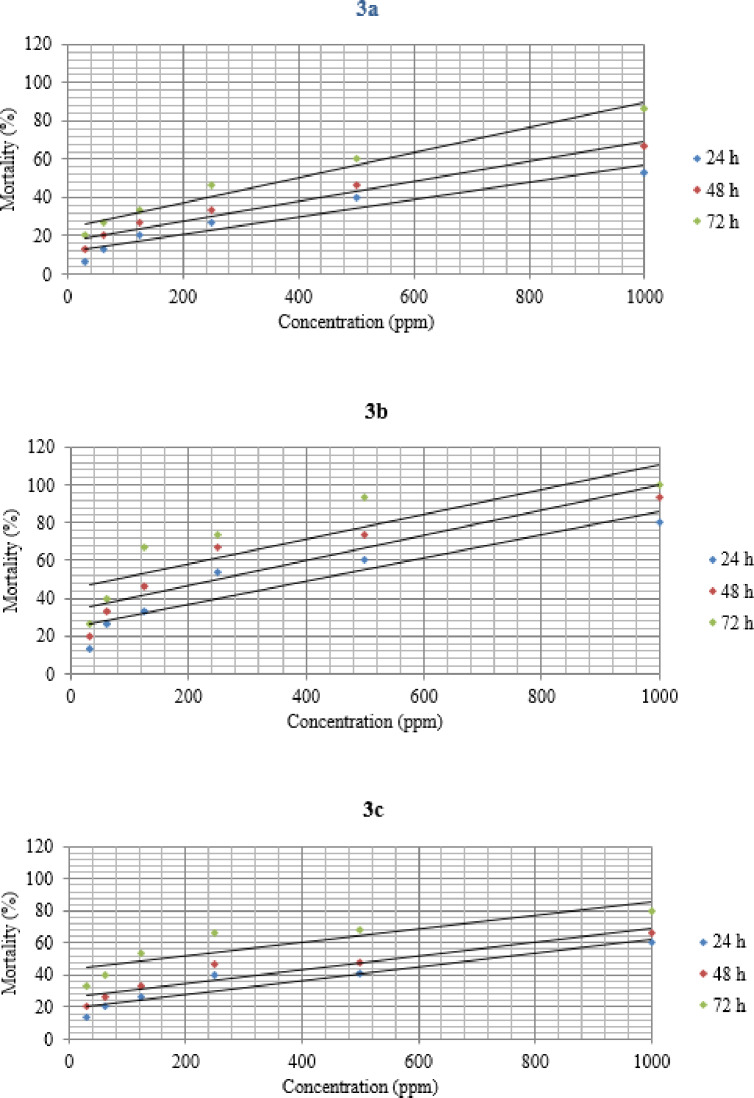
Mortality analysis of *T. castaneum* (adult) to concentratio–response.

Contact toxicity assays of benzofuran derivatives on *T. castaneum* adults shown in Table 3 revealed that **3b** was the most effective compound, followed by **3c** and **3a**, with LC_50_ values of 242.46 (141.37–417.72), 634.21 (224.35–1870.94), and 877.24 (318.17–2510.53) ppm at 24 h, 131.81 (81.87–211.53), 392.54 (156.63–1014.37), and 508.51 (223.07–1189.1) ppm at 48 h, and 79.58 (51.63–122.03), 106.37 (46.96–236.57), and 226.17 (130.82-392.48) ppm at 72 h, respectively. The tested *T. castaneum* populations exhibited a range of slopes for probit response curves, spanning from 0.83 to 1.79 for compounds **3a**, **3b**, and **3c**. In contrast, compounds **1**, **3d**, **3e**, **3f** and **5** exhibited very low toxicity (i.e., LC_50_ values greater than 1000 ppm) or no contact toxicity against *T. castaneum* over time. The survival rate of the treated insects gradually decreased over time, with significant variations noted across different periods. The data presented in Table 4 show the mean mortality % of T. *castaneum* beetles that have been exposed to benzofuran-pyrazole compounds at varied doses for 24, 48, and 72 h. The mortality rate at 1000 ppm was significantly higher than at 31.25 ppm after 24, 48, and 72 h of exposure. The mortality caused by **3b** was 93.33% until 48 h post-treatment, then reached 66.67% after 48 h of exposure for both tested compounds **3a** and **3c** at 1000 ppm, while **3b** caused 100% mortality at the end of 72 h, which was followed by 86.67% mortality caused by **3a** and 80% mortality caused by **3c**. This data reveals that compound **3b**, at a level of 1000 ppm, possessed the highest average mortality rate after 72 h. Moreover, Fig. 3**’s** concentration–response curve depicts he percentage of those who died due to explosion to benzofuran derivatives.

**Table 3:** The lethal concentrations of Benzofuran derivatives againste *T. castaneum* (adult) at laboratory conditions.

Compounds	Time (h)	LC_5_ (F.L)	LC_50_ (F.L)	LC_95_ (F.L)	Slope ± SE	*P*	Chi-square
**3a**	24	21.18 (4.07–102.72)	877.24 (318.17–2510.53)	36,332.14 (1734.30)	1.02 ± 9.6	1	0.07
	48	10.65 (1.55–66.95)	508.51 (223.07–1189.1)	24,276.1 (1484.6–455,764.4)	0.98 ± 0.08	0.98	0.44
	72	9.19 (1.87–42.44)	226.17 (130.82–392.48)	5563.92 (1021.3–32,486.46)	1.18 ± 0.08	0.83	1.48
**3b**	24	10.72 (2.39–45.46)	242.46 (141.37–417.72)	5482.71 (1054.2–30,436.95)	1.21 ± 0.08	0.98	0.40
	48	8.95 (2.34–32.71)	131.81 (81.87–211.53)	1941.23 (617.09–6358.8)	1.41 ± 0.09	0.96	0.64
	72	9.62 (3.21–27.84)	79.58 (51.63–122.03)	658.45 (301.41–1471.89)	1.79 ± 0.13	0.87	1.22
**3c**	24	7.38 (0.65–73.83)	634.21 (224.35–1870.94)	54,485.27 (1357.5–2,709,634)	0.85 ± 0.08	0.98	0.41
	48	3.26 (0.16–57.11)	392.54 (156.63–1014.37)	47,281.49 (1072.3–2,647,428)	0.97 ± 0.07	0.98	0.44
	72	1.11 (3.38–29.67)	106.37 (46.96–236.57)	10,194.49 (660.3–186,671.3)	0.83 ± 0.08	0.99	0.33

LC, Lethal concentrations; F.L, Fiducial limits; SE, Standard error; P, Probability.

**Table 4:** Mortality (%) ± SE of Benzofuran derivatives against *T. castaneum* (adult) at laboratory conditions.

Compounds	Time (h)	Concentrations (ppm)					
		31.25	62.5	125	250	500	1000
**3a**	24	6.67 ± 0.33	13.33 ± 0.33	20.00 ± 0.00	26.67 ± 0.33	40.00 ± 0.00	53.33 ± 0.33
	48	13.33 ± 0.33	20.00 ± 0.58	26.67 ± 0.33	33.33 ± 0.33	46.67 ± 0.33	66.67 ± 0.33
	72	20.00 ± 0.00	26.67 ± 0.33	33.33 ± 0.33	46.67 ± 0.33	60.00 ± 0.00	86.67 ± 0.33
**3b**	24	13.33 ± 0.33	26.67 ± 0.33	33.33 ± 0.33	53.33 ± 0.33	60.00 ± 0.00	80.00 ± 0.00
	48	20.00 ± 0.00	33.33 ± 0.33	46.67 ± 0.67	66.67 ± 0.67	73.33 ± 0.33	93.33 ± 0.33
	72	26.67 ± 0.33	40.00 ± 0.00	66.67 ± 0.33	73.33 ± 0.33	93.33 ± 0.33	100.00 ± 0.00
**3c**	24	13.33 ± 0.33	20.00 ± 0.00	26.67 ± 0.33	40.00 ± 0.00	41.00 ± 0.00	60.00 ± 0.00
	48	20.00 ± 0.00	26.67 ± 0.33	33.33 ± 0.33	46.67 ± 0.33	47.67 ± 0.00	66.67 ± 0.33
	72	33.33 ± 0.33	40.00 ± 0.00	53.33 ± 0.33	66.67 ± 0.33	68.67 ± 0.33	80.00 ± 0.00

### Insecticidal activity and structure activity relationship (SAR) studies

**Fig. 4:**
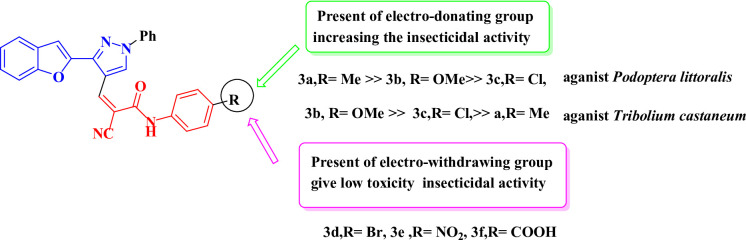
SAR of Benzofuran–pyrazol-acrylamide derivatives as potent insecticides.

The newly synthesized chemicals’ insecticidal properties rely on their electrical environment and structural framework. Figure 4 Our research indicates that the in vitro inhibitory activity of 4- phenyl substituted Benzofuran–pyrazol-acrylamide **3a–f** is higher than that of 4- thiazole substituted at compound **5**. From this it is explained, the effect of functional group on the phenyl which the para-substituent of the phenyl ring at position 4 **(3b** > **3c** > **3a**), the electron-donating group (methyl or methoxy chlorine), increases the insecticidal activity in Benzofuran–pyrazol-acrylamide. In contrast to the other groups **3d**,** 3e**,** 3f** withdrawing group (bromo, nitro, carboxylic group) in the para-substituent of the phenyl ring at position 4 in the pyrazole no contact toxicity against *T. castaneum* over time. And in the end it becomes clear, the toxicity against *Spodoptera littoralis* and *Tribolium castaneum* increasing by electron donating at para position of phenyl in Benzofuran–pyrazol-acrylamide derivative.

### DFT calculation

The study of chemical compounds and their reactivity is fundamental to understanding their behaviour in various chemical reactions. One of the key factors influencing the properties and reactivity of compounds is their stereochemistry, specifically the configuration of substituents around double bonds, which is represented by E/Z isomerism. The exploration of the relationship between molecular structure and reactivity is crucial for the rational design of new molecules with desired properties, particularly in fields such as organic synthesis, drug design, and material science. The Gaussian09w software was employed to investigate the influence of the molecular structure of the synthesized compounds on their electronic properties and reactivity^43,44^; a certain number of system properties can be obtained *via* DFT, without resorting to wave functions. A DFT calculation was conducted to determine the relative stability of the ***E*** and ***Z*** forms in support of the stereochemical assignment. As a result of optimized geometries and energy values, it can be determined which configuration is energetically preferable, which provides a molecular-level rationale for selecting the most stable configuration. As a consequence, stereochemistry can be assigned more robustly and complements experimental spectral data. In addition, the DFT calculation amount is relatively small. As a result, the orbital was calculated using the B3LYP basis set. The reactivity of a molecule can be evaluated based on the energies of its highest occupied and lowest unoccupied molecular orbitals (E_HOMO_ and E_LUMO_). A number of theoretical parameters were then determined, such as the energy gap (ΔE), hardness (η), and overall softness (S), through the following equations^45^

$$\:{\Delta\:}\mathrm{E}=\:{E}_{LUMO}-\:{E}_{HOMO}$$.

χ is the negative of µ. Parr et al. A calculated η is determined using a finite difference approximation with ionization energy and electron affinity as follows:

$$\:{\upeta\:}=\:\frac{IE-EA}{2}$$

It follows that $\:{E}_{LUMO}$ and $\:{E}_{HOMO}$ are equivalent representations of IE and EA, respectively, according to Koopman’s theorem, that is, $\:-\:{E}_{LUMO}\:=\:IE$ and $\:-{E}_{HOMO}\:=\:EA$. Therefore, η can be expressed as:

$$\:{\upeta\:}=\frac{{E}_{LUMO}-{E}_{HOMO}\:}{2}\:$$

The value of S is the inverse of η and is expressed as:

$$\:\mathrm{S}=\:\frac{1}{{\upeta\:}}$$

The molecular structures of the investigated compounds, along with their corresponding ***E*** and ***Z*** stereoisomers, are presented in Table 5. A visual representation of the compounds facilitates interpretation of the computed electronic properties and relative stabilities by providing a clear visual reference.

**Table 5:**
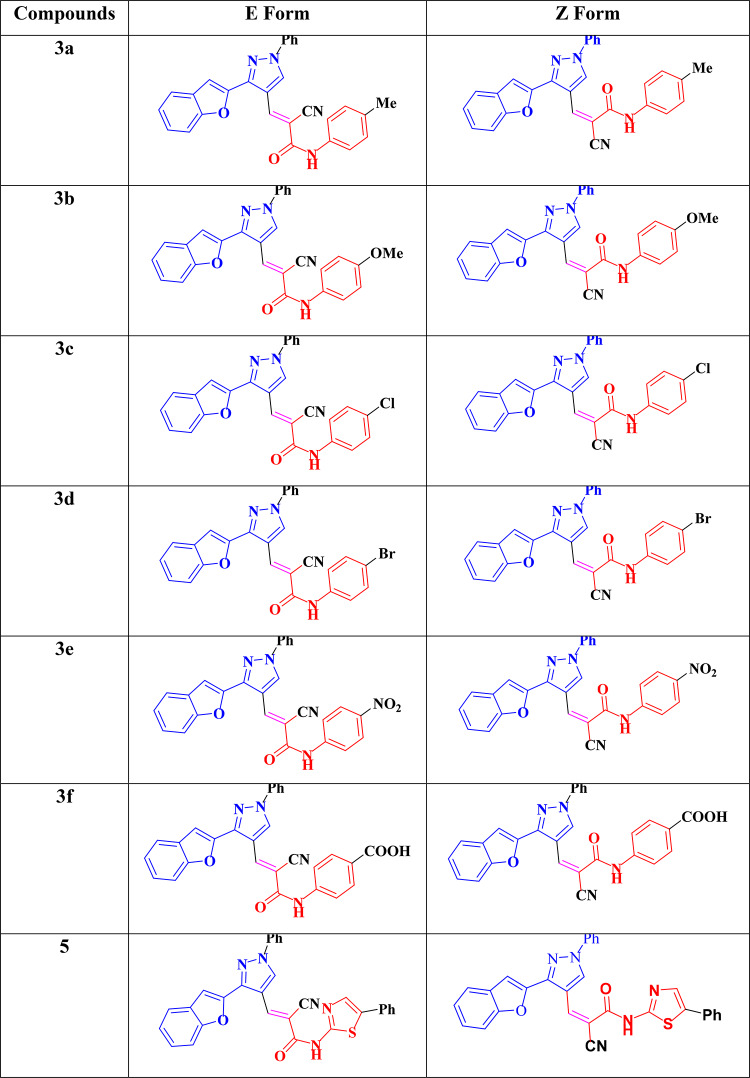
Compounds studied using DFT in ***E*** and ***Z*** Forms.

The comparative quantum chemical analysis of the investigated compounds in their ***E*** and ***Z*** configurations reveals pronounced differences in electronic structure, global reactivity descriptors, and dipole moments. Within each pair, the stereoisomeric arrangement strongly influences the energy gap (ΔE), global hardness (η), softness (S), chemical potential (µ), and global electrophilicity index (ω). These differences directly reflect changes in the spatial distribution of substituents and frontier orbitals, thereby modulating the electronic reactivity and adsorption propensity of the molecules^46^.

For compounds **3a**,** 3c**,** 3d**, and **3e**, the *E* forms consistently exhibit smaller ΔE values and higher softness compared to their *Z* counterparts. This indicates that *E* configurations are generally more electronically reactive and better at facilitating charge transfer interactions with metallic surfaces. The case of ***3c.E*** is especially interesting because its ΔE is only 0.464 eV, which gives it a very high softness (S ≈ 4.31 eV⁻¹) and a strong electrophilicity index. In contrast, ***3c.Z*** undergoes a drastic increase in ΔE (3.147 eV), rendering it far less reactive. Such a sharp *E*/*Z* disparity suggests that steric and electronic factors jointly govern the stabilization of the frontier orbitals, with the E form maintaining a favourable orbital alignment for electron exchange^47^.

This electronic profile strongly supports the identification of ***3c.E*** as the most reactive species, since the combination of an exceptionally small energy gap, high softness, and elevated electrophilicity is widely recognized as a hallmark of enhanced molecular reactivity and interaction capability.

In contrast, compounds **3b**, **3f**, and **5** show an opposite tendency, where the *Z* form possesses slightly reduced ΔE values and enhanced softness compared to the *E* form. These differences, although more subtle than in compound **3c**, suggest that the *Z* configuration in these systems promotes a better overlap of frontier orbitals or alleviates intramolecular repulsion, leading to improved electronic reactivity. Additionally, the *Z* forms of these molecules tend to exhibit higher dipole moments, which could enhance electrostatic interactions at polar interfaces or metal–solution boundaries. When all compounds are compared crosswise, ***3c.E*** emerges as the most electronically active candidate by far, with a uniquely small energy gap and extreme softness. It is followed by ***3e.E***, which also demonstrates low hardness, high softness, and the highest dipole moment (~ 10 D) among the entire set, indicating a strong potential for electrostatic adsorption on metallic surfaces. ***5.Z*** and ***3f.Z*** also ranks highly in softness and electrophilicity, making them competitive *Z* forms with promising reactivity. On the other hand, compounds such as ***3d.Z*** and ***3a.Z*** exhibit larger energy gaps and lower softness, implying a reduced electronic contribution to molecular reactivity and interaction capability. Importantly, this electronic reactivity trend is consistent with the molecular docking results, where the most reactive E forms, particularly ***3c.E*** display enhanced binding affinity toward acetylcholinesterase (AChE), a well-established biological target for insecticidal agents. The superior electronic characteristics of ***3c.E*** likely facilitate stronger noncovalent interactions within the AChE active site, thereby supporting its proposed mode of action. The global electrophilicity index (ω) further refines this assessment, as higher values reflect an increased tendency to accept electron density.

The order of electrophilicity strongly supports the exceptional role of ***3c.E***, which far exceeds the rest, while ***3e.E***, ***5.Z***, and ***3f.Z*** form a second tier of highly electrophilic molecules. In practical terms, the ***E*** configuration of compound 3c can be considered the most electronically reactive species, with its electronic structure finely tuned for efficient charge donation and acceptance, supporting strong intermolecular interactions. Overall, the stereoisomeric effect is decisive: in some systems (compound **3c**), the *E/Z* switch completely transforms the electronic behaviour, while in others (compounds **3b**, **3f**, and **5**), the differences are more nuanced but still significant. These findings highlight the importance of considering stereochemistry when designing compounds for optimal binding interactions with biological targets. From a structure–activity perspective, the pronounced superiority of ***3c.E*** rationalizes its prioritization as the most reactive and biologically relevant candidate in the present study. The combined evaluation of ΔE, softness, electrophilicity, and dipole moment suggests that ***3c.E*** and ***3e.E*** should be prioritized for further studies of molecular interactions and reactivity, while ***5.Z*** and ***3f.Z*** represent the most promising ***Z*** configurations for practical applications^48^.

**Fig. 5:**
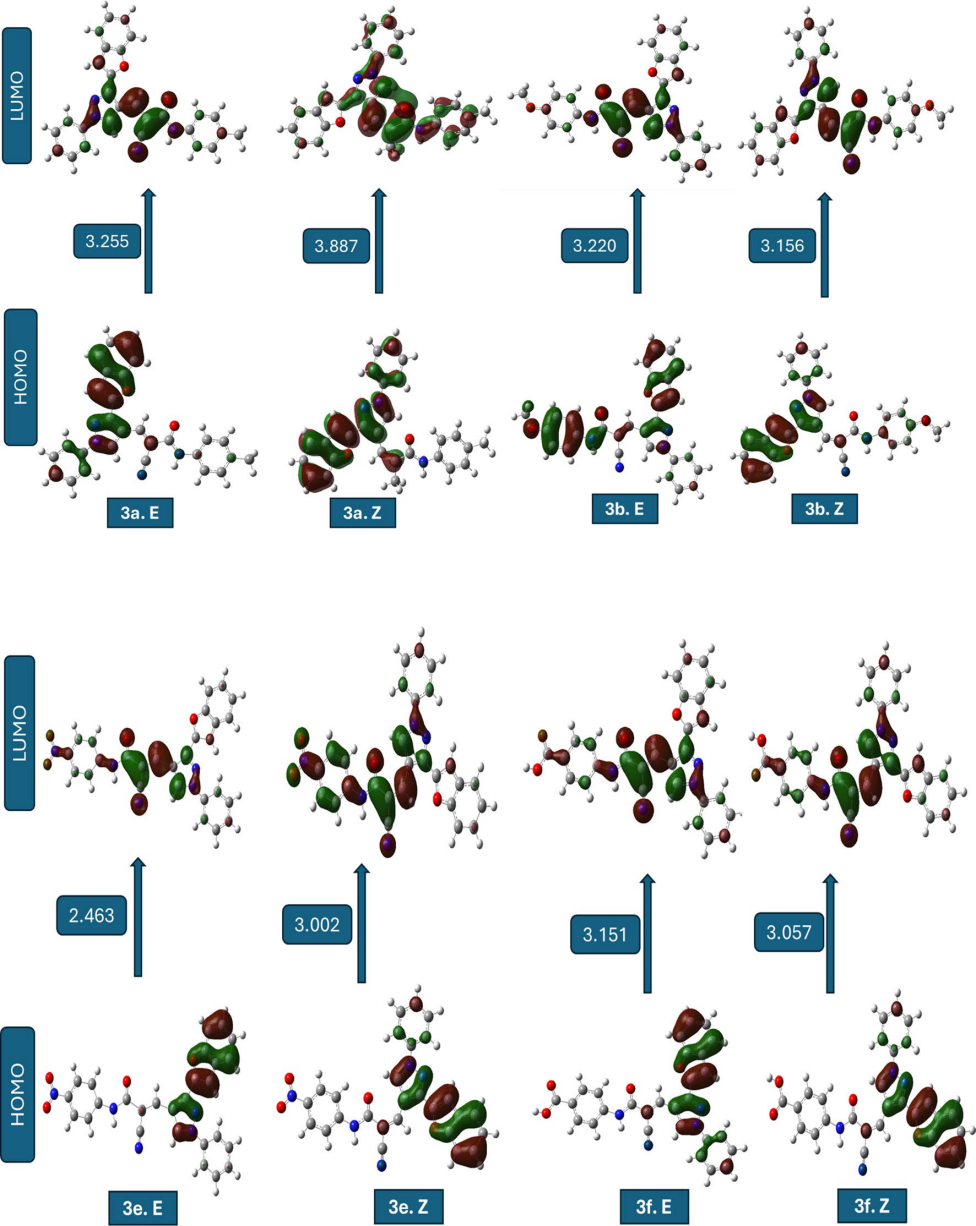

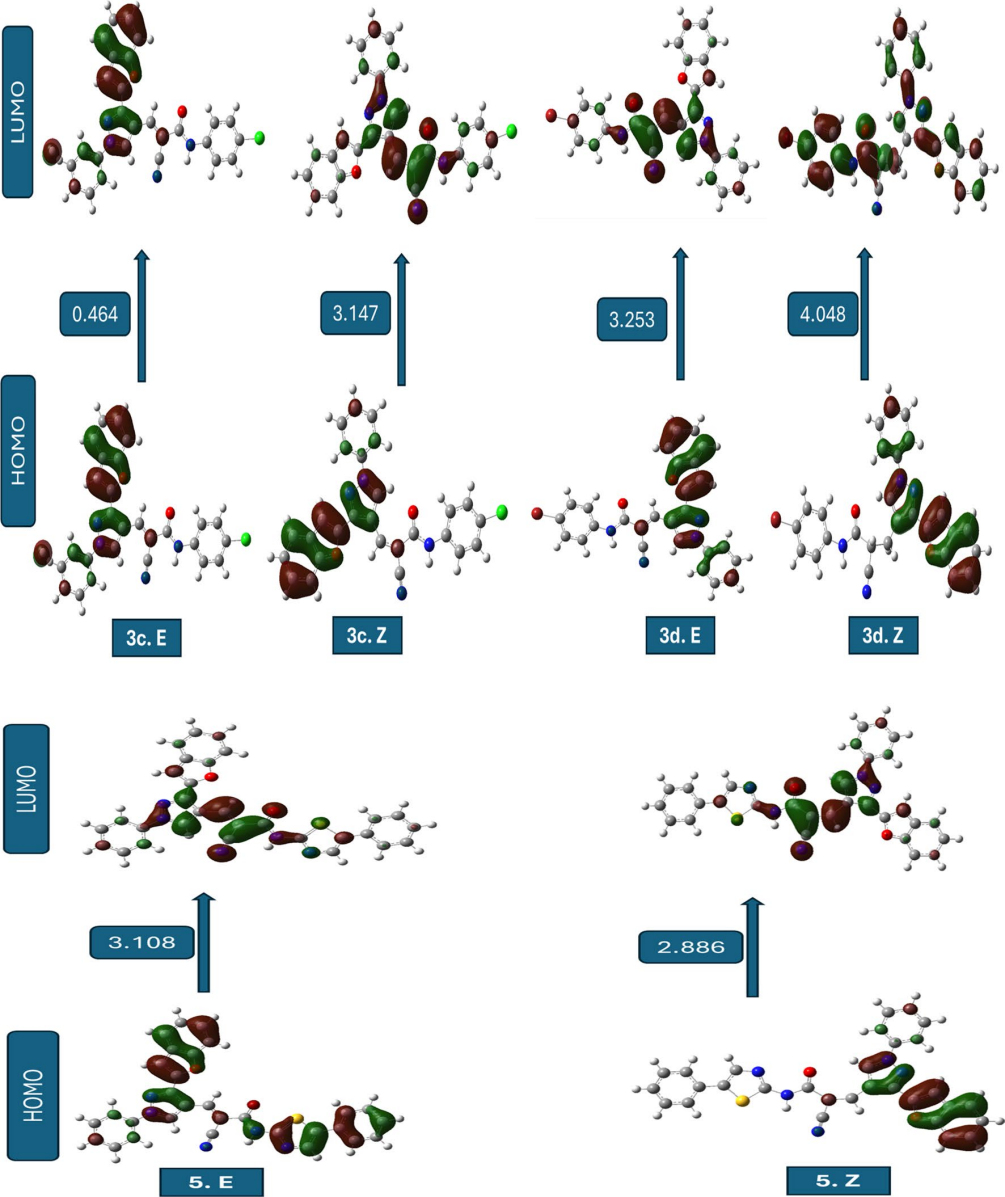
The pictures of optimized structures, HOMO, and LUMO of compound molecules.

The frontier molecular orbital (FMO) analysis highlights the strong influence of stereochemistry on electronic reactivity. In compounds **3a-e**, the ***E*** forms generally show superior HOMO delocalization and better HOMO–LUMO overlap, resulting in smaller ΔE and higher softness, with ***3c.E*** emerges as the most reactive species due to its exceptionally favorable orbital alignment. On the other hand, compounds 3f and 5 prefer the Z configuration, which has a wider HOMO and LUMO delocalization across the conjugated skeleton. This makes donor–acceptor interactions stronger. Figure 5; Table 6 summarize these findings, presenting visual and tabulated data that further elucidate the trends in electronic properties and reactivity across the various compounds. Overall, the results confirm that stereoelectronic effects are system-dependent: the ***E*** form dominates in compounds **1–5**, while the ***Z*** form is electronically superior in **6** and **7**. The optimized geometries (Fig. 5) of the studied compounds (***3a-f.E***–***5.E*** and ***3a-f.Z***–***5.Z***) confirmed that all structures correspond to true energy minima without imaginary frequencies. The comparison between forms indicated that the ***E***-forms are generally more stable than the ***Z***-forms due to reduced steric hindrance and more favourable spatial orientation of substituents. The ***E/Z*** configurations of the synthesized compounds were not assigned solely on the basis of experimental spectral data. Although the ¹H NMR spectra confirmed the formation of the conjugated acrylamide framework, clear diagnostic coupling constants required for unambiguous ***E/Z*** discrimination were not observed, likely due to signal overlap and extended conjugation effects. Consequently, the stereo chemical assignment was primarily supported by DFT geometry optimization and relative energy calculations. All optimized structures corresponded to true energy minima with no imaginary frequencies, and the most energetically stable configuration for each compound was considered the preferred stereoisomer.

**Table 6:** Calculated quantum chemical parameters for the studied compounds (eV).

Compound	E_HOMO_	E_LUMO_	ΔE Energy gap	$\:\boldsymbol{\eta}\:=\:\boldsymbol{\varDelta\:}\boldsymbol{E}\:/2$ global hardness	$\:\boldsymbol{S}\:=\:1/\:\boldsymbol{\eta}$ global softness	µ chemical potential	Dipole moment
**3a.** ***E***	− 5.533	− 2.278	3.255	1.628	0.614	− 3.906	2.4064
**3a.** ***Z***	− 5.218	− 1.331	3.887	1.944	0.515	− 3.275	3.2445
**3b.** ***E***	− 5.457	− 2.237	3.220	1.610	0.621	− 3.847	1.5399
**3b.** ***Z***	− 5.472	− 2.316	3.156	1.578	0.634	− 3.894	3.3201
**3c.** ***E***	− 5.828	− 5.364	0.464	0.232	4.310	− 5.596	1.3643
**3c.** ***Z***	− 5.528	− 2.381	3.147	1.574	0.636	− 3.955	4.8090
**3d.** ***E***	− 5.633	− 2.380	3.253	1.627	0.615	− 4.007	4.7298
**3d.** ***Z***	− 5.292	− 1.244	4.048	2.024	0.494	− 3.268	1.9645
**3e.** ***E***	− 5.502	− 3.039	2.463	1.232	0.812	− 4.271	9.9972
**3e.** ***Z***	− 5.665	− 2.663	3.002	1.501	0.666	− 4.164	7.0446
**3f.** ***E***	− 5.612	− 2.461	3.151	1.576	0.635	− 4.037	5.3067
**3f.** ***Z***	− 5.534	− 2.477	3.057	1.529	0.654	− 4.006	6.3375
**5.** ***E***	− 5.581	− 2.473	3.108	1.554	0.644	− 4.027	2.6062
**5.** ***Z***	− 5.518	− 2.632	2.886	1.443	0.693	− 4.075	3.7518

**Fig. 6:**
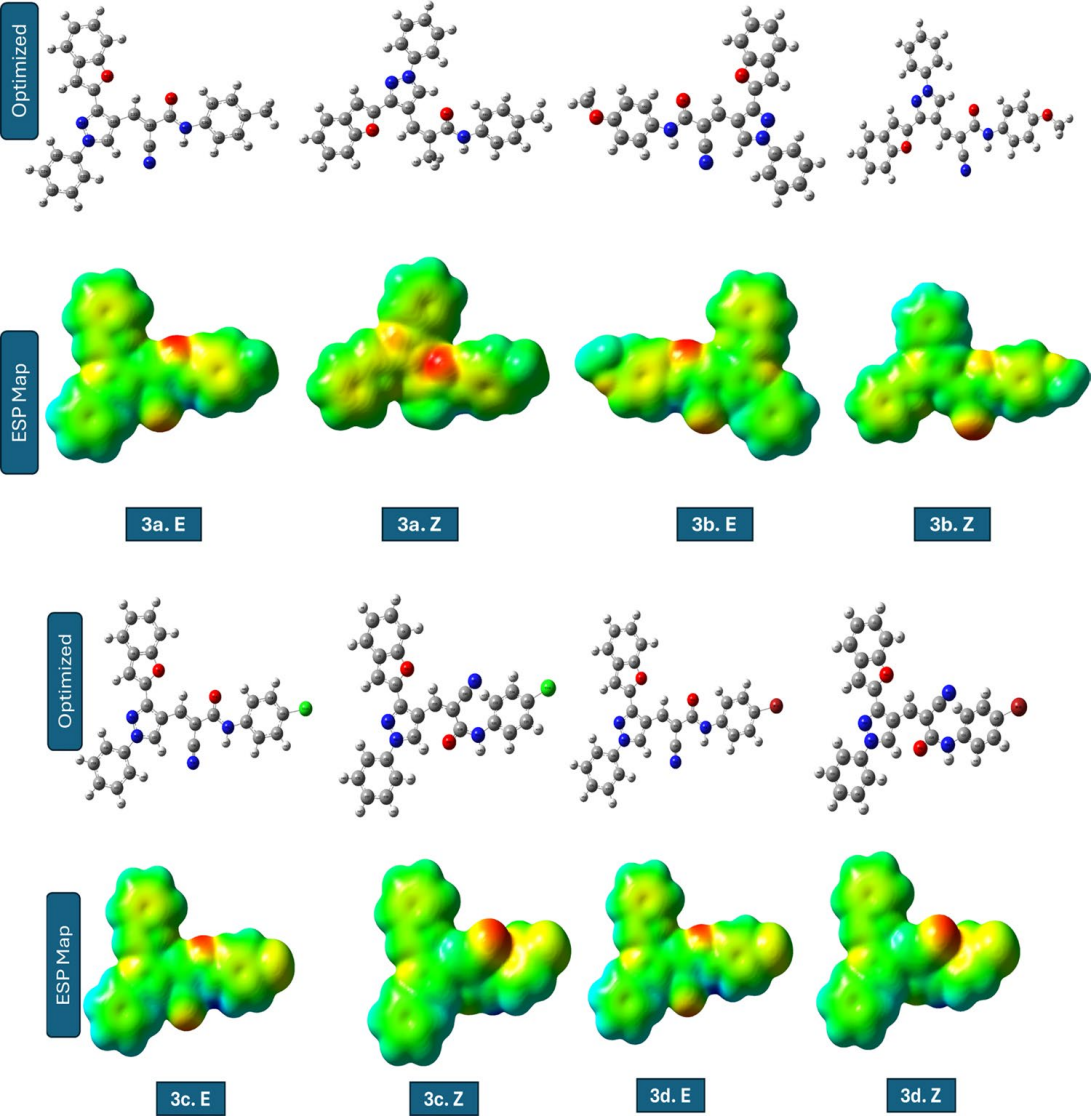

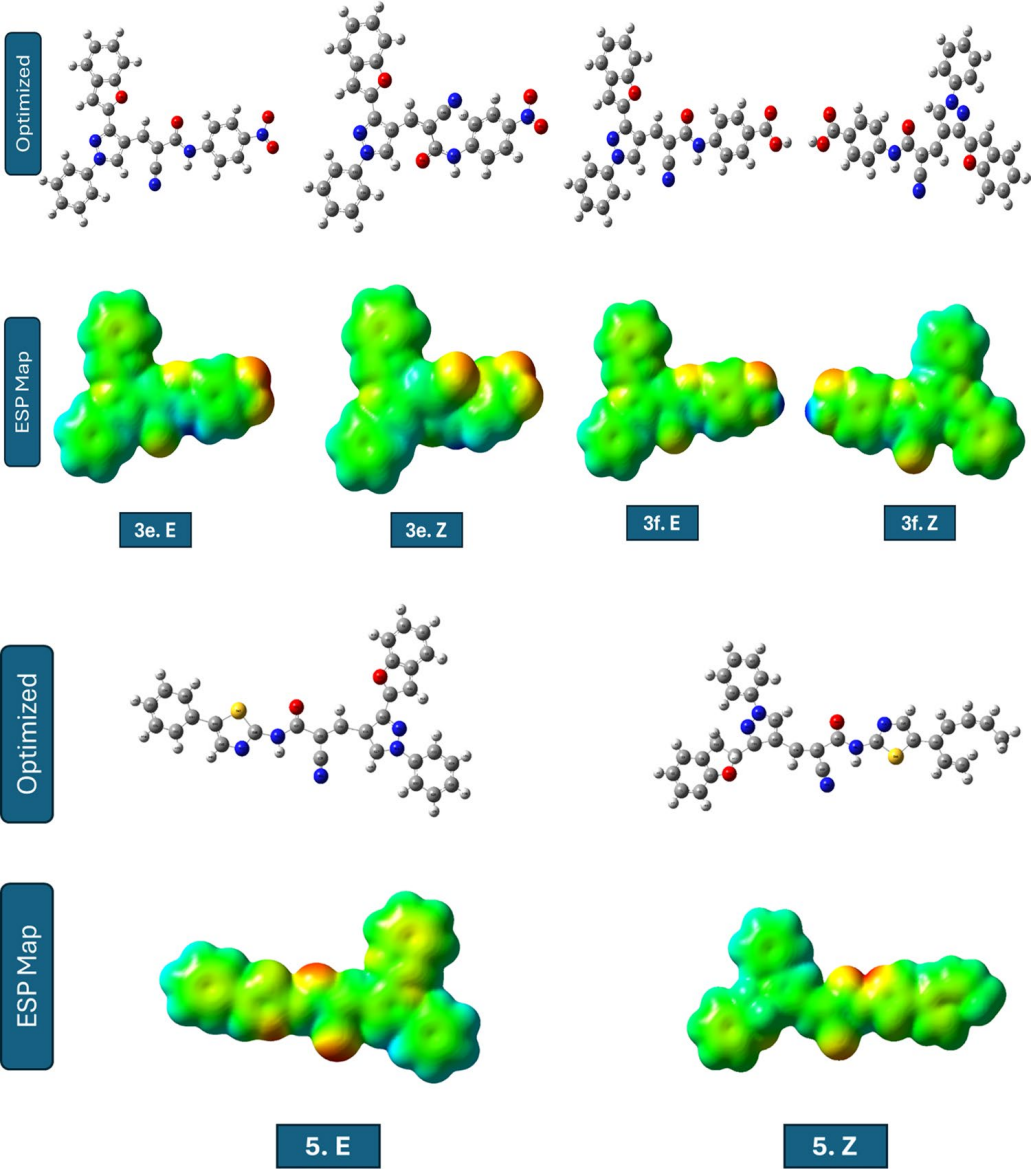
Optimized structures and electrostatic potential (ESP) maps of the studied compounds.

Electrostatic potential (ESP) maps revealed electron-rich regions mainly around heteroatoms and electron-deficient regions near hydrogen atoms attached to electron-withdrawing groups. The geometrical difference between *E* and *Z* configurations significantly affects the ESP distribution: E-foems display a clearer separation of positive and negative potential regions, which could enhance directional intermolecular interactions such as hydrogen bonding, while *Z*-forms show closer proximity and overlap of electron-rich zones, influencing intramolecular contacts and potentially altering their reactivity. Overall, *E*-forms exhibit higher thermodynamic stability and more defined polarity patterns, whereas *Z*-forms may offer unique electronic features relevant for specific interaction pathways (Fig. 6).

### Molecular Docking

#### Molecular Docking of selected molecules with *Spodoptera Litura* (AChE) receptor

Table 7 details the molecular interactions of the same set of compounds **3a**, **3b**, and **3c**, positive control carbaryl, and the co-crystallized inhibitor NAF with the acetylcholinesterase (AChE) enzyme from *Spodoptera litura*, a major agricultural pest known as the tobacco cutworm or common cutworm. This enzyme plays a vital role in terminating nerve impulses by hydrolyzing acetylcholine, and its inhibition disrupts pest nervous systems, making it a prime target for insecticides. For compound **3a**, a single hydrophilic hydrogen bond is formed with Thr5 at 2.23 Å, likely involving a polar group like the amide or ether oxygen from its structure (methyl-substituted benzofuran-indazole hybrid). Hydrophobic interactions include pi-alkyl with Phe9 (4.05 Å), pi-sigma with Val313 (3.50 Å), and pi-sigma with Asn350 (3.93 Å), resulting in 1 hydrogen bond and 4 total bonds, with a binding affinity of -8.5 kcal/mol, the strongest among the novel ligands. This suggests efficient anchoring in the hydrophobic pocket, with Thr5 possibly near the oxyanion hole, stabilizing the ligand for catalytic interference. Compound **3b**, featuring a carboxylic acid group, forms one hydrophilic hydrogen bond with Asn350 at 2.48 Å, which could involve the acid’s carbonyl or hydroxyl engaging the asparagine’s amide. Hydrophobic contacts are more numerous: pi-alkyl with Ile310 (4.36 Å) and Phe9 (4.18 Å), pi-stacked with Phe9 (4.50 Å), and pi-cation with Lys430 (4.30 Å), yielding 1 hydrogen bond and 5 total bonds, with an affinity of − 8.50 kcal/mol. The acid group might introduce some repulsion or suboptimal orientation in the *Spodoptera* AChE site compared to Tribolium, reducing affinity slightly. In compound **3c**, with its chloro substituent enhancing lipophilicity, no hydrophilic hydrogen bonds are reported, but three hydrophobic interactions dominate: pi-alkyl with Lys6 (5.35 Å), Val313 (4.40 Å), and Val8 (3.88 Å), leading to 0 hydrogen bonds, 3 total bonds, and an affinity of -8.00 kcal/mol. This purely hydrophobic binding mode suggests the chlorine facilitates halogen-pi or van der Waals interactions in the acyl pocket, compensating for the lack of polar contacts.

**Fig. 7:**
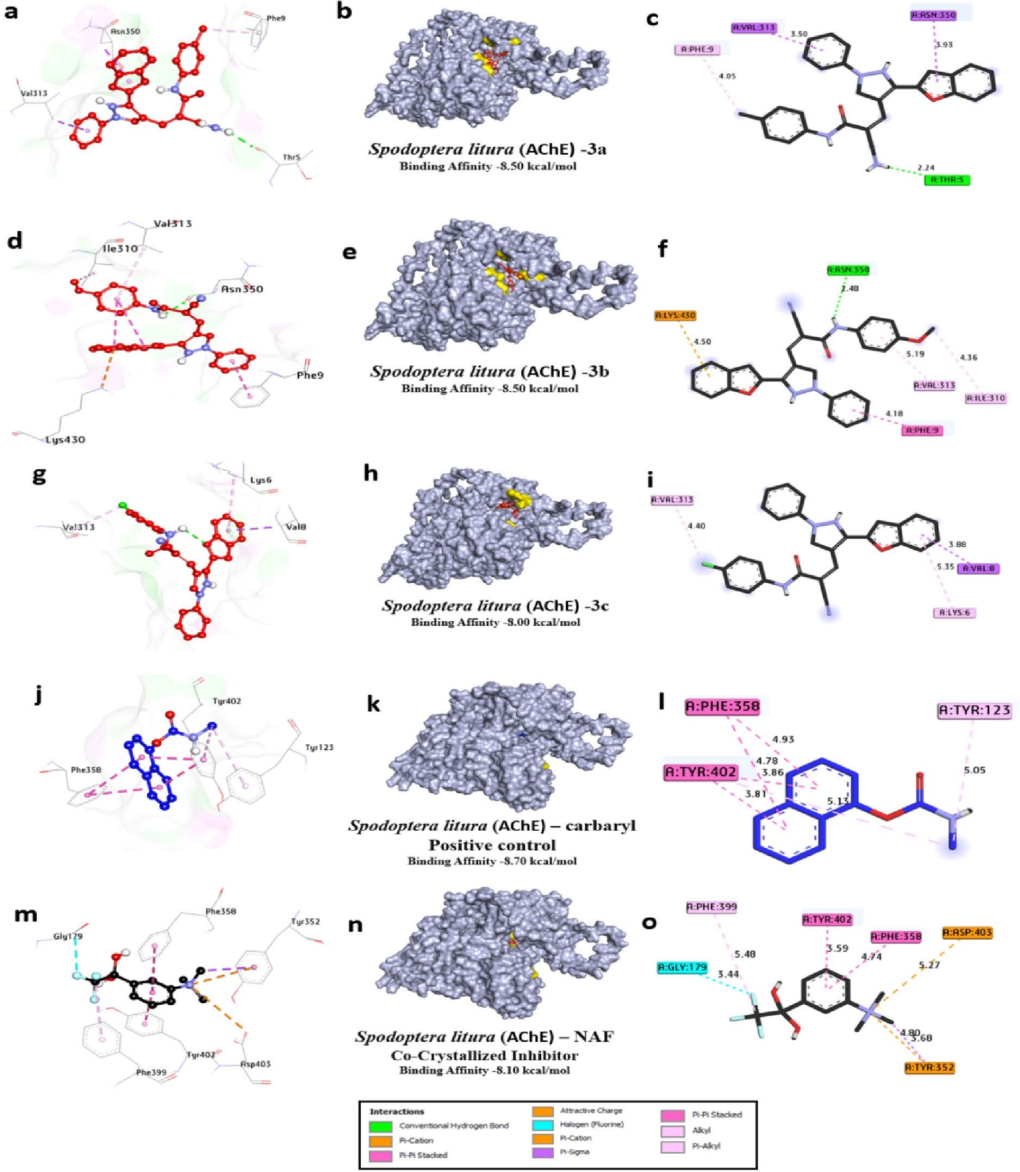
3D representations of compounds **3a**, **3b** and **3c** conformations at the binding pockets of ***Spodoptera litura*** **(AChE)** protein: (**a–c**) 3a, (**d–f**) 3b, (**g–i**) 3c, (**j–l**) carbaryl (Positive control) and (**m–o**) NAF negative ligand.

Compared to **3a** and **3b**, the reduced bond count correlates with similar affinity, implying quality over quantity in interactions. The positive control carbaryl, a known carbamate insecticide, lacks hydrophilic bonds in this docking but forms six hydrophobic interactions: pi-alkyl with Tyr123 (5.05 Å), Tyr402 (5.13 Å), Phe358 (4.93 Å), and Phe399 (5.48 Å); pi-stacked with Phe358 (4.78 Å) and Tyr402 (3.81 Å); and pi-pi stacked with Tyr402 (3.85 Å), resulting in 0 hydrogen bonds, 6 total bonds, and − 8.7 kcal/mol affinity. This strong binding likely arises from the carbamate mimicking the substrate, positioning for potential covalent modification of the catalytic site. Lastly, the co-crystallized NAF inhibitor exhibits no hydrophilic bonds but seven hydrophobic ones: pi-alkyl with Phe399 (5.48 Å), Gly179 (3.44 Å), and Phe358 (4.74 Å); halogen with Tyr402 (3.59 Å); pi-stacked with Phe358 (4.27 Å); pi-cation with Asp403 (5.27 Å) and Tyr352 (4.80 Å); and pi-sigma with Tyr352 (3.68 Å), with 0 hydrogen bonds, 7 total bonds, and − 8.10 kcal/mol affinity. The fluorinated nature enables unique halogen bonds, enhancing specificity in the active site. Overall, the novel ligands **3a**, **3b**, and **3c** demonstrate competitive affinities to controls, relying on conserved hydrophobic residues like Phe9, Val313, and Asn350 in *Spodoptera* AChE, which align with the acyl and anionic subsites. These results position the compounds as promising leads for species-specific or broad pest control agents. These in-silico findings are consistent with recent studies El-Saghier et al., 2023^49^, which also report potent *Spodoptera* AChE inhibition, underscoring the potential of these compounds as inhibitor agents (Table 7; Fig. 7).

**Table 7:**
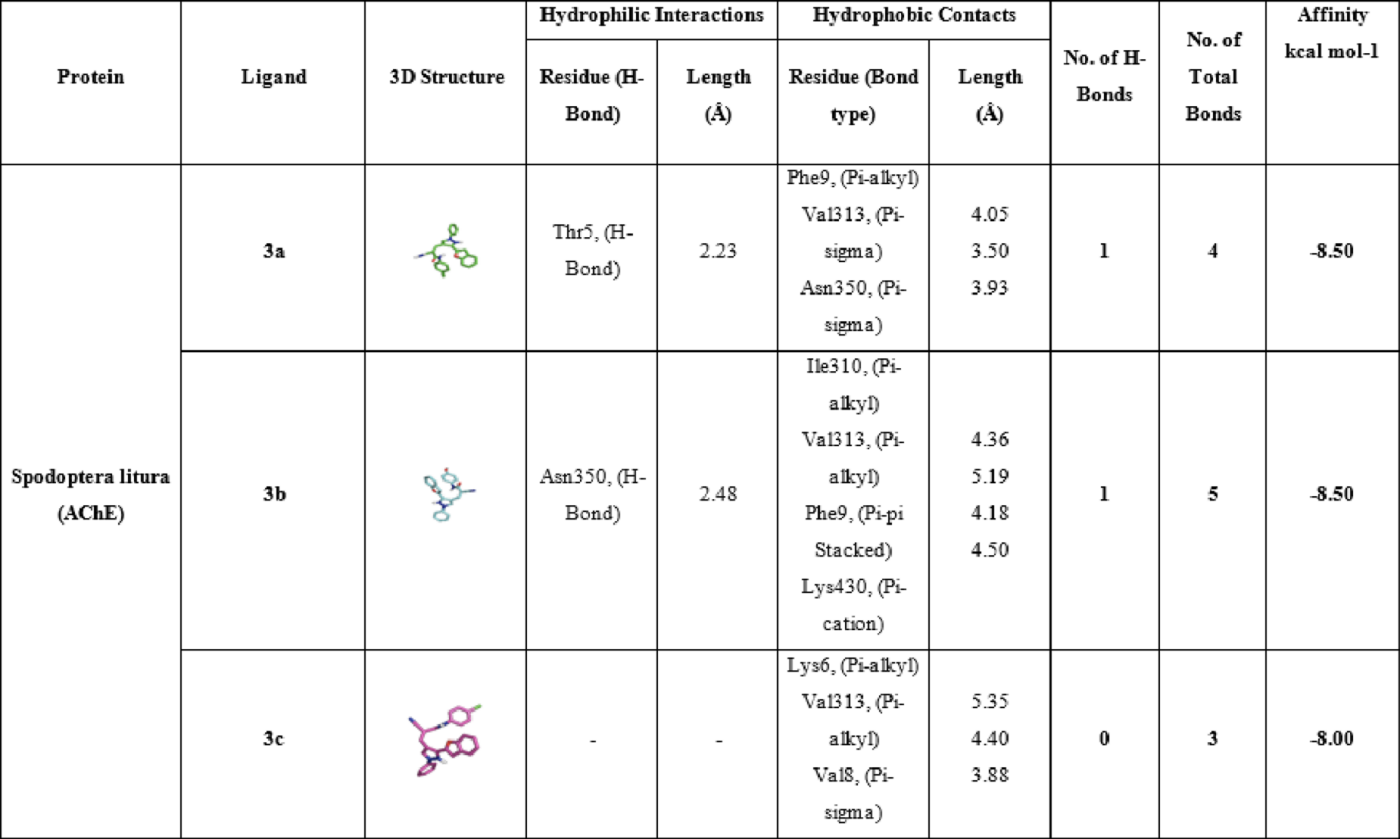

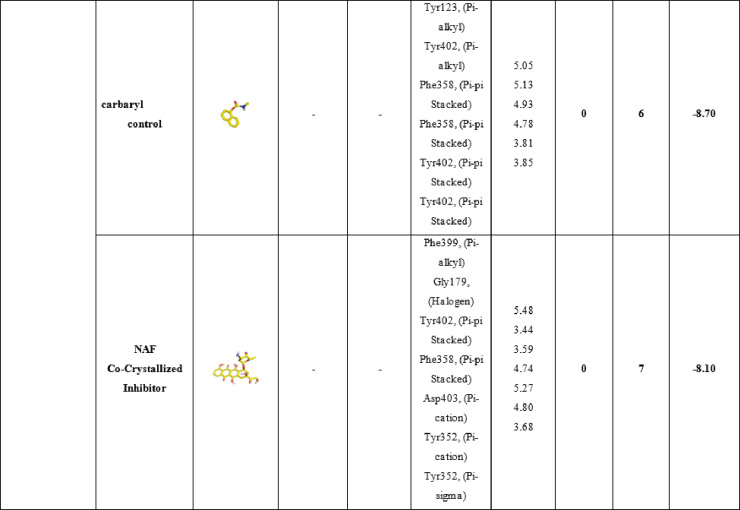
Molecular interactions of ligands with *Spodoptera Litura* (AChE) protein:

#### Molecular Docking of selected molecules with *Tribolium castaneum* (AChE) receptor

Table 8 shows the molecular interactions between three compounds (**3a**, **3b**, and **3c**), the positive control compound carbaryl, the co-crystallized inhibitor NAF, and the acetylcholinesterase (AChE) enzyme of *Tribolium castaneum*, also known as the red flour beetle. Inhibition of the acetylcholinesterase enzyme is an important mode of action for many different classes of insecticides, all of which target the function of this key nervous-system enzyme that breaks down acetylcholine. The binding affinities for all compounds are quite strong, ranging from − 7.8 to − 8.5 kcal/mol, which is comparable to the binding affinities for known inhibitors such as carbaryl, − 8.5 kcal/mol, and NAF, − 8.3 kcal/mol. Compound **3a** engages in two hydrogen bonds: one with Phe216, which is 2.50Å, and the other with Ser213, which is 2.13Å. The hydrophobic bonds include pi-alkyl bonds with Pro224, which is 4.80Å, with Val357, which is 5.39Å; with Val360, which is 5.48Å; and with Arg218, which is 5.01Å. Other bonds include a pi-sigma bond with Val357, which is 4.05Å; and a pi-stacked bond with Val357, which is 4.28Å. The two hydrogen bonds and six bonds complete the binding affinity of − 8.2 kcal/mol. The presence of Ser213, which is part of the catalytic triad, may indicate the inhibition of the serine hydroxyl function critical in catalysis, while the six pi-alkyl bonds with valine residues indicate van der Waals interactions in the hydrophobic pocket of the active site. Compound **3b** has one hydrophilic hydrogen bond with Glu85 at 3.08 Å, in addition to eight hydrophobic bonds, including pi-alkyl bonds with Val357 (4.05 Å, 4.95 Å, 5.19 Å), Pro224 (5.19 Å), Val360 (3.81 Å), and Arg358 (4.42 Å), as well as a carbon-hydrogen bond with Glu298 (3.68 Å), contributing one hydrogen bond and eight bonds. This results in its binding affinity being − 8.8 kcal/mol. Compound **3c** engages in one hydrophilic hydrogen bond at 3.08 Å with Glu85 and eight hydrophobic interactions analogous to ligand 3b: pi-alkyl interactions with Val360 (5.01 Å, 5.49 Å, and 4.14 Å), Val357 (4.11 Å), Pro224 (5.30 Å and 4.42 Å), and Arg218 (4.15 Å).

**Fig. 8:**
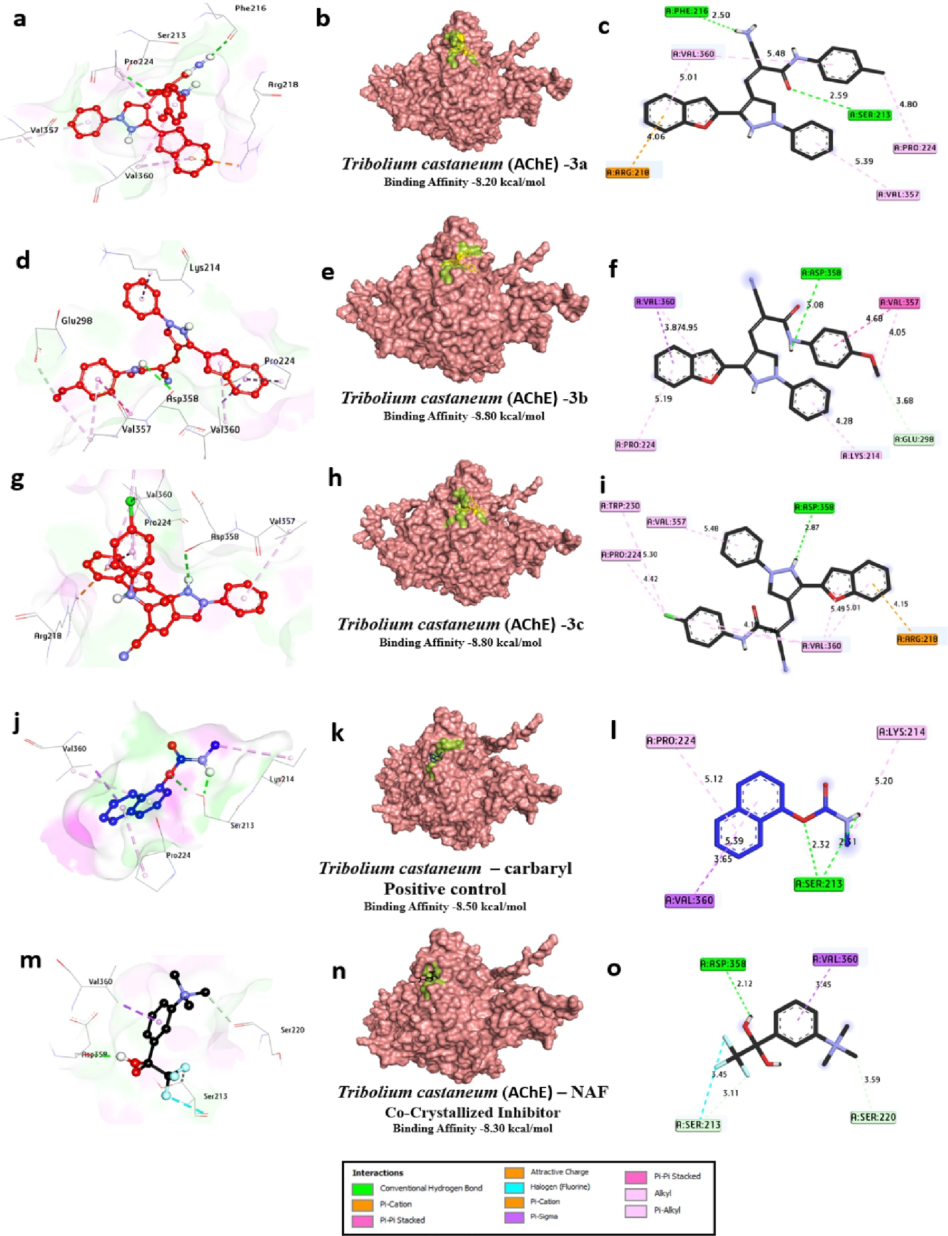
3D representations of compounds **3a**, **3b** and **3c** conformations at the binding pockets of *Tribolium castaneum* (AChE) protein: (**a–c**) 3a, (**d–f**) 3b, (**g–i**) 3c, (**j–l**) carbaryl (Positive control) and (**m–o**) NAF negative ligand.

This conformation provides one hydrogen bond, eight bonds, and − 8.5 kcal/mol. Such critical residues in ligands like Val360, Val357, Pro224, Glu85, Trp230, and Arg218 indicate ionic interactions/polar forces important for stabilizing ligands within the AChE anionic site. The positive control, carbaryl, makes two hydrophilic hydrogen bonds with Ser213 (2.32 Å and 2.31 Å) and six hydrophobic interactions: pi-alkyl with Lys214 (5.20 Å), Pro224 (5.12 Å), and Val360 (5.39 Å); a pi-sigma interaction with Pro224 (3.65 Å); and a halogen interaction with Ser213 (3.45 Å). Its two hydrogen bonds and six total bonds give carbaryl the highest binding affinity of − 8.5 kcal/mol. The carbamate group in Carbaryl produces covalent-like bonds with Ser213, in line with the binding affinity of carbamate insecticides. The co-crystallized inhibitor NAF engages in one hydrogen bond with Asp358 (2.12 Å) and five hydrophobic interactions, including a halogen bond with Val360 (3.45 Å), a pi-sigma bond with Ser220 (3.59 Å), and carbon-hydrogen bonds with Ser213 (3.11 Å). NAF has one hydrogen bond and five total bonds, resulting in an affinity value of − 8.3 kcal/mol. By comparison, the new compounds **3a**, **3b**, and **3c** have binding affinities comparable to the controls, mainly based on hydrophobic interactions that mainly target the peripheral anionic site and the acyl pocket, including conserved active site residues like Val357, Val360, and Pro224. The hydrogen bonds to either Ser213 or Glu85 imply interference with the catalytic activity. These findings suggest that the compounds 3a, 3b, and 3c have potential as AChE inhibitors in insect control. These in-silico findings are consistent with recent studies (Reyes-Espinosa et al., 2024; Gholivand et al., 2017)^50,51^, which also report potent AChE inhibition, underscoring the potential of these compounds as insect cholinesterase inhibitors (Table 8; Fig. 8).

**Table 8:**
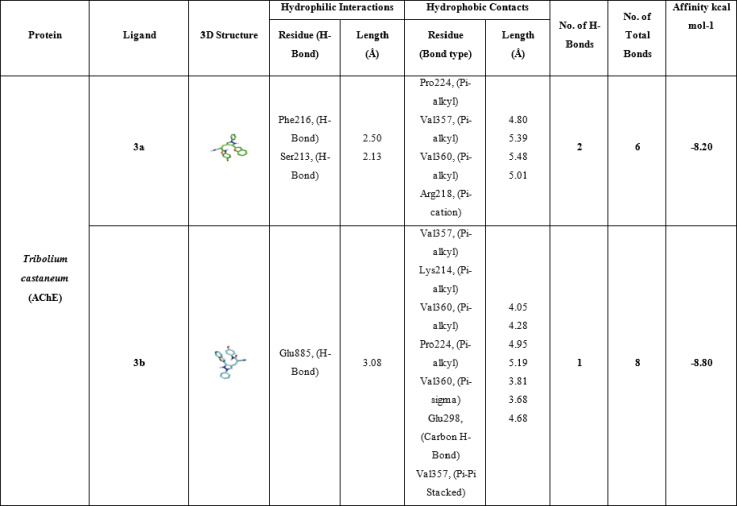

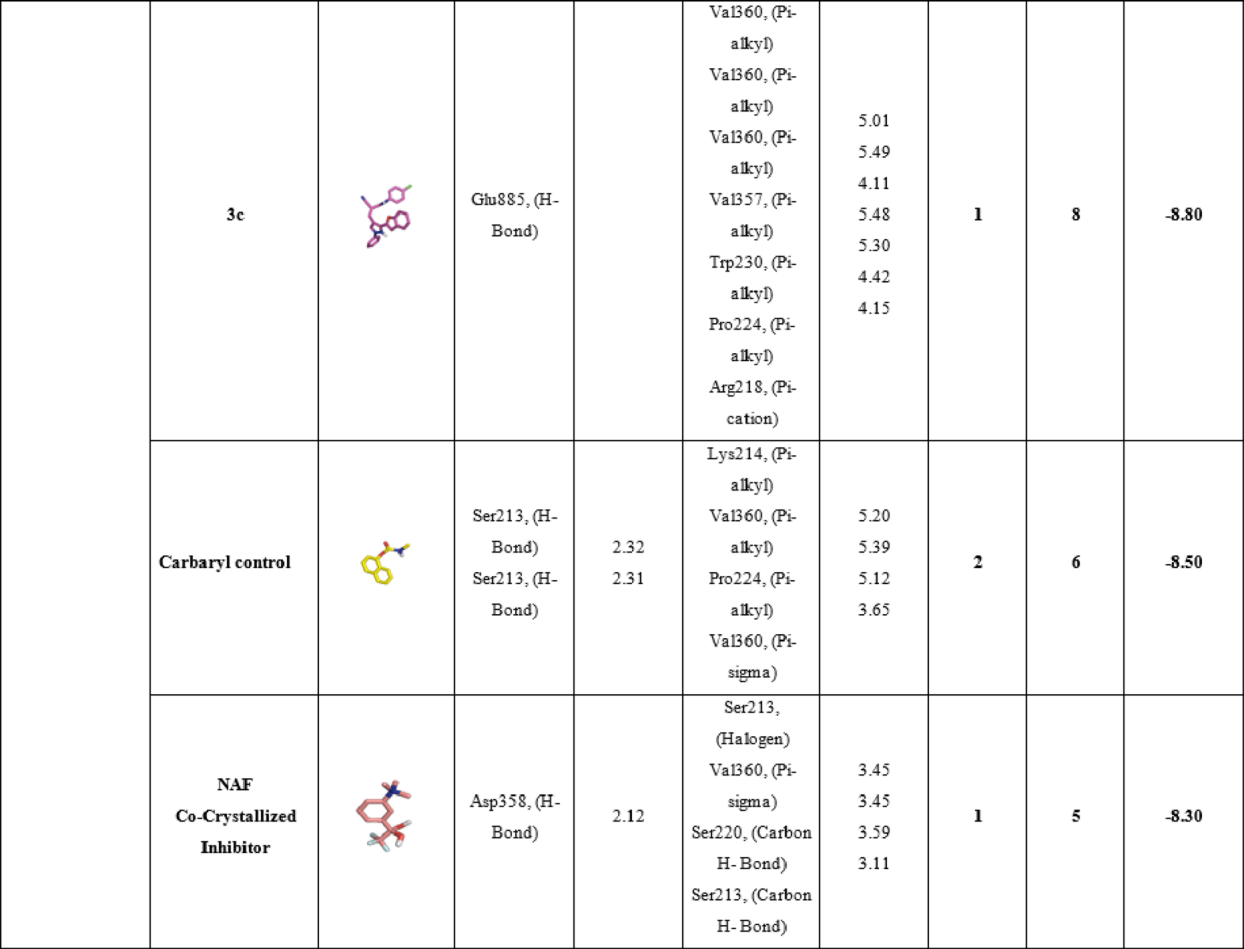
Molecular interactions of ligands with *Tribolium castaneum* (AChE) protein:

#### In Silico pharmacokinetics ADMET prediction of synthesized compounds

**Fig. 9:**
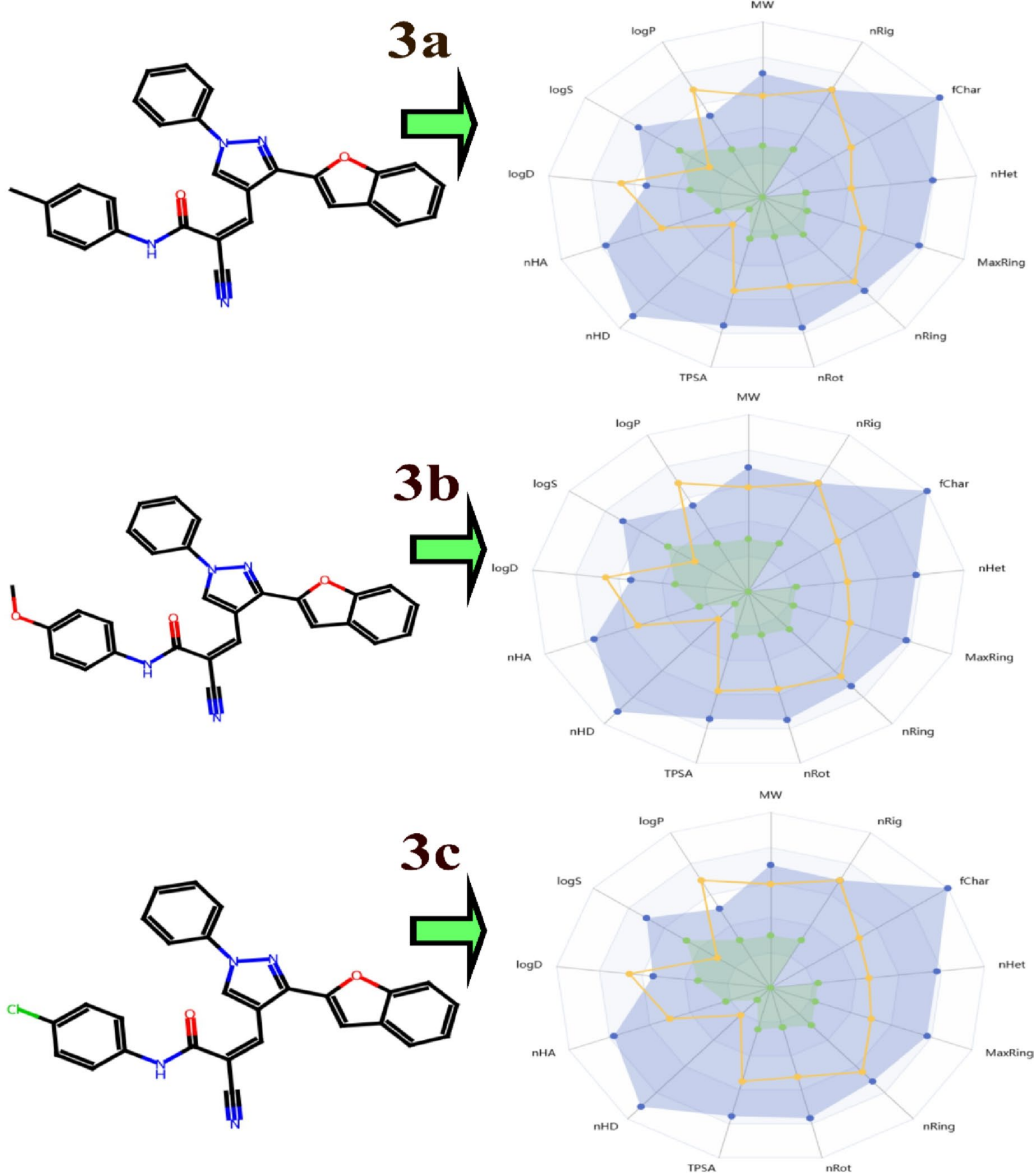
Oral bioavailability analysis of compounds using ADMETlab 2.0. (**A**) Depicts the chemical structure of compounds **3a**, **3b** and **3c**. (**B**) Shows its bioavailability radar plot, graphically summarizing key physicochemical properties governing absorption.

Table 9; Fig. 9 show predicted pharmacokinetics and physicochemical properties for three compounds, **3a**, **3b**, and **3c**.On physiochemical properties, molecular weights (MW) of all five molecules fall within the range between approximately 444 to 460 for compound **3a** with a weight of 444.16 following by; MW of 460.15 for compound -b combined with the highest energy gap; while compound c exhibits virtually a similar highest energy at MW calculated at around 464.10 are deemed small molecules suitable for further drug-like applications since they fall within the typically defined rule of thumb limit threshold (350–500 Da) for oral bioavailability compared to other structures. Their volumes (Vol) are comparable, ranging from 469.45 Å^3^ for 3a to 478.24 Å³ for **3b**, suggesting similar steric bulk. Hydrogen bond acceptors (nHA) and donors (nHD) are low, with nHA at 6.00 for **3a** and **3c**, and 7.00 for **3b**, while nHD is 1.00 for all, which implies moderate polarity but limited hydrogen bonding capacity that could influence solubility and membrane permeability. Topological polar surface area (TPSA) values are 83.85 Å² for **3a** and **3c**, and 93.08 Å² for **3b**, all below the 140 Å² threshold often associated with good oral absorption. Rotatable bonds (nRot) are 5.00–6.00, indicating flexibility that might aid in conformational adaptation for binding but could also increase metabolic liability. Heteroatom counts (nHet) are 6.00–7.00, and formal charges (fChar) are neutral (0.00), while flexibility (Flex) is around 0.30–0.44, suggesting semi-rigid molecules. Stereocenters (nStereo) are absent (0.00), and logS (aqueous solubility) ranges from − 7.25 to -6.68, indicating poor water solubility that may necessitate formulation strategies. LogD (distribution coefficient at pH 7.4) is 4.15–5.37, showing lipophilicity favorable for passive diffusion across membranes but potentially risking accumulation in fatty tissues. LogP (partition coefficient) is 5.32–5.66, reinforcing their hydrophobic nature.

Parameters of Table 9 related to absorption: HIA values are positive (indicating 0.0014 to 0.0060 probability of poor absorption, meaning the likelihood of high absorption is high), with F (20%) and F (30%) values having low bioavailability at these doses (− 0.0148 to 0.0252 F(20%): indicative of low or variable levels of absorption). Caco-2 permeability values appear negative (− 5.1877 to − 5.0954), possibly exhibiting low transportation levels through the intestinal cells, which may be the weakest link in the enteric forms of these compounds. MDCK permeability values remain low (4.9454–4.9252). Caco-2 permeability values give negative values (− 5.1877 to − 5.0954) of P-glycoproteins (Pgp), showing the limited level of transportation of these compounds through the cells of the human intestine, which may be the weakest link in the enteric forms of these compounds. Substrate and inhibition values of MDCK-MDR-1 cells’ P-glycoproteins: Substrate values for 3a, 3b: Non-substrate compounds give values of 0.0000 to 0.0004. Potential inhibitors give values of 0.9999 to 1.0000. Similarly, values of 3c compounds follow the same pattern. Cell Fraction unbound in Plasma Blood-Brain Barrier Penetration.

In the case of the metabolic profile given in the Table 9, it has been predicted that all the compounds tested can be substrates for the different isoforms of cytochrome P450: CYP1A2 inhibitors (0.9341–0.9986, non-inhibitors), CYP2C19 inhibitors (0.2978–0.6978, mixed), CYP2C9 inhibitors (0.8001–0.9047, inhibitors), CYP2D6 (0.0007–0.0718, non-inhibitors/substrates), and CYP3A4 inhibitors/substrates (0.5898–0.7580). This suggests the possibility of drug interactions due to the inhibition of cytochromes, with special emphasis on CYP2C9 and CYP3A4, as the major pathways for drug metabolism. The excretion parameters suggest a total clearance (CLtot) of about 1.51–3.46 mL/min/kg, with the value of the renal clearance (Rc) being negligible (0.00), thus proposing the possibility of hepatic clearance. The toxicity predictions showed negative values for the AMES mutagenicity (0.3677–0.6077) and positive values for hERG inhibition I and II, with the possibility of cardiotoxicity (0.2704–0.5538). Other toxicities like carcinogenicity (0.5461–0.6007), eye corrosion/injury (0.0007–0.7537), and respiratory toxicity (0.5124–0.7971) vary, with neurotoxicity and ototoxicity being low to moderate.

In addition, Table 10 shows the toxicity risk, in addition to some physicochemical properties, for the same compounds. The toxicities are generally negative (−) for mutagenic, tumorigenic, irritant, and reproductive toxicities, showing good toxic properties. The oral toxicities, using CLop (consensus logP), are estimated to be 4.79 for compound **3a**, 4.38 for compound **3b**, and 5.06 for compound **3c**. The solubility (logS) values range from − 6.48 to − 6.87, possibly showing some solubility problems. Drug-likeness scores are very low (0.32–0.35), and the drug scores are extremely low (0.03–0.35) when considering ideal drug-likeness values, possibly showing some anomalies related to ideal drug-like physicochemical properties according to Lipinski’s Rule of Five, where all compounds are within the rule but show some values in logP that are higher than 5 units in some cases.

**Table 9:** Prediction of pharmacokinetics and physicochemical properties of compounds.

Id	ID	3a	3b	3c	Id	ID	3a	3b	3c
Physicochemical properties	MW	444.16	460.15	464.10	Metabolism	CYP1A2-inh	0.9341	0.9530	0.9986
	Vol	469.45	478.24	467.36		CYP1A2-sub	0.2299	0.6225	0.0354
	Dense	0.95	0.96	0.99		CYP2C19-inh	0.9978	0.9921	0.9999
	nHA	6.00	7.00	6.00		CYP2C19-sub	0.0004	0.0156	0.0022
	nHD	1.00	1.00	1.00		CYP2C9-inh	0.9801	0.9690	0.9869
	TPSA	83.85	93.08	83.85		CYP2C9-sub	0.0047	0.0334	0.0185
	nRot	6.00	7.00	6.00		CYP2D6-inh	0.0007	0.0011	0.0275
	nRing	5.00	5.00	5.00		CYP2D6-sub	0.0718	0.4539	0.0074
	MaxRing	9.00	9.00	9.00		CYP3A4-inh	0.6898	0.9335	0.2047
	nHet	6.00	7.00	7.00		CYP3A4-sub	0.7590	0.1032	0.9048
	fChar	0.00	0.00	0.00	Excretion	LM-human	0.9151	0.8961	0.7667
	nRig	30.00	30.00	30.00		cl-plasma	3.2460	4.1187	2.9687
	Flex	444.16	460.15	464.10	Toxicity	t0.5	0.6460	0.5940	0.7081
	nStereo	469.45	478.24	467.36		BCF	1.1628	1.2421	1.3428
Solubility	LogS	− 7.2517	− 6.6815	− 7.4861		IGC50	4.4761	4.4666	4.7690
	LogD	4.1517	4.1305	4.0538		LC50DM	5.4339	5.7567	6.3169
	LogP	5.3270	4.7828	5.6925		LC50FM	5.5447	5.7408	6.0706
	ESOL Log S	− 6.61	− 6.37	− 6.90		hERG	0.2704	0.3108	0.3961
	Ali Log S	− 7.64	− 7.42	− 7.91		hERG-10 μm	0.5558	0.5210	0.6650
	Silicon-IT class	Poorly	Poorly	Poorly		DILI	0.9609	0.9792	0.9824
Drug-likeness	Lipinski Rule	Accepted	Accepted	Accepted		Ames	0.6077	0.6761	0.4773
	Pfizer Rule	Accepted	Accepted	Accepted		ROA	0.3862	0.3704	0.4597
	Golden Triangle	Accepted	Accepted	Accepted		FDAMDD	0.8446	0.8461	0.8581
Absorption	Pgp-inh	0.9999	0.9994	0.9999	Toxicophore rules	SkinSen	0.9347	0.7867	0.9501
	Pgp-sub	0.0000	0.0000	0.0000		Carcinogenicity	0.5461	0.6668	0.4754
	HIA	0.0014	0.0254	0.0006		EC	0.0007	0.0004	0.0003
	F (20%)	0.0060	0.0110	0.0005		EI	0.7537	0.5995	0.5339
	F (30%)	0.0148	0.0252	0.0044		Respiratory	0.6124	0.7076	0.5404
	Caco-2	− 5.1877	− 5.0964	− 5.1436		H-HT	0.7971	0.7610	0.7893
	MDCK	− 4.9454	− 4.9252	− 4.9800		Neurotoxicity-DI	0.2353	0.2753	0.2642
Distribution	BBB	0.0017	0.0002	0.0190	Medicinal chemistry	Ototoxicity	0.3051	0.3270	0.3456
	PPB	98.9022	98.8528	99.3787		QED	0.261	0.254	0.238
	VDss	0.1089	0.1102	− 0.2031		Synth	2.404	2.415	2.414
	Fu	0.5848	0.6963	0.2684		Fsp3	0.036	0.036	0

**Table 10:** Prediction of toxicity risks and oral toxicity prediction results of compounds.

No	Ligand	Toxicity risks				Physicochemical properties					
		Mutagenic	Tumorigenic	Irritant	Reproductive	CLogP	Solubility	Molecular weight	TPSA	Drug likeness	Drug score
1	**3a**	(−)	(−)	(−)	(−)	4.79	− 6.48	444.0	83.85	0.72	0.32
2	**3b**	(−)	(−)	(−)	(−)	4.38	− 6.15	460.0	93.08	0.88	0.35
3	**3c**	(−)	(−)	(−)	(−)	5.06	− 6.87	464.0	83.85	2.82	0.33

#### Molecular dynamics simulation (MDS)

**Fig. 10:**
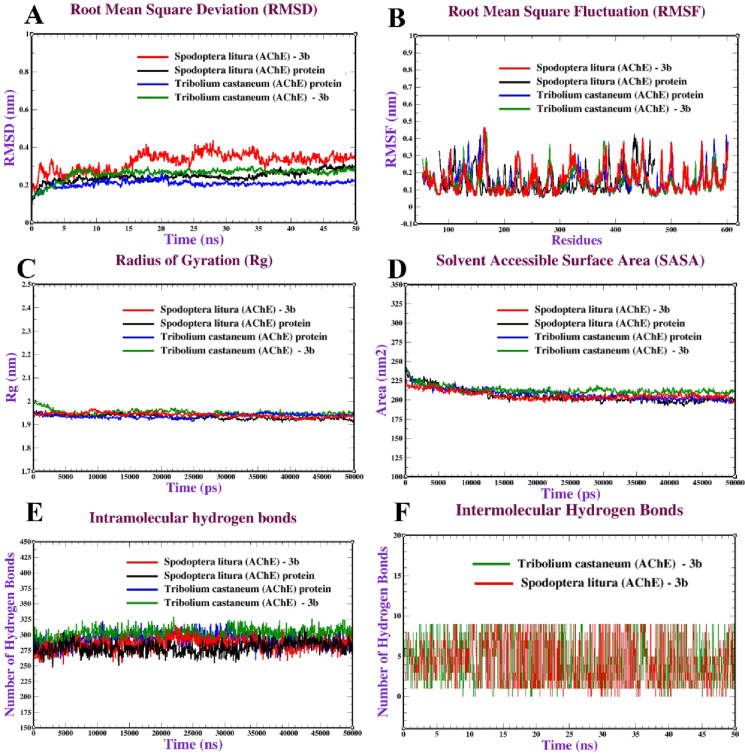
Molecular dynamics of *T. castaneum* (AChE) and *S. litura* (AChE) proteins (free and **3b**-bound): (**A**) RMSD, (**B**) RMSF, (**C)** Radius of gyration (Rg), (**D**) SASA, (**E**) Intramolecular hydrogen bonds and (**F**) Intermolecular hydrogen bonds.

Molecular dynamics (MD) simulations were performed on various systems, including the *T. castaneum* (AChE) and *S. litura* (AChE) proteins alone and their complex with compound (**3b**). The structural stability of these protein-ligand complexes and their free forms was evaluated by analyzing the root mean square deviation (RMSD) over a 50 ns simulation period. The RMSD analysis evaluates the deviations of protein backbone atoms from their initial configurations, with values settling between 0.10 and 0.45 nm after initial fluctuations, showing structural constancy with time. *T. castaneum* (AChE) (free and **3b**-bound) had an RMSD ranging from 0.15 to 0.3 nm, which stabilized after about 40 ns, showing strong structural integrity with minor conformational changes. In contrast, for *S. litura* (AChE) proteins (free and **3b**-bound), the RMSD ranged from 0.20 to 0.45 nm, stabilizing after roughly 35 ns, demonstrating strong structural flexibility with conformation changes. The **3b**-bound AChE had slightly higher RMSD values than the free equivalent, indicating minimal structural changes during ligand binding. Overall, all systems demonstrated acceptable stability, with RMSD values remaining below 0.45 nm, suggesting that binding of 3b does not significantly alter protein structures over 50 ns (Fig. 10A). Root mean square fluctuation (RMSF) analysis revealed the flexibility of individual residues during the simulations. Complexed states AChE (free and 3b-bound) exhibited higher variations (up to 0.50 nm) than free states, indicating increased local mobility, most likely due to ligand interactions. RMSF values in all systems (free and 3b-bound) remained below 0.5 nm, with occasional peaks up to 0.50 nm in specific residues, indicating localized flexibility, particularly in loop areas (Fig. 10B). The radius of gyration (Rg) was utilized to determine the compactness of protein structures, which indicates their overall shape stability. Rg values ranged from 1.95 to 2.00 nm across all systems, indicating stable conformations in both free and bound states. Rg values in the 3b-bound *S. litura* (AChE) system (free and bound) remained between 1.95 and 2.02 nm, indicating a constant compact structure. The complex’s Rg remains lower and more constant, implying that ligand binding promotes a more compact and stable folded form. The 3b-bound *T. castaneum* (AChE) system (free and 3b-bound) revealed somewhat higher Rg values, with a modest increase after 20 ns, indicating a slight structural expansion due to 3b binding (Fig. 10C). Solvent accessible surface area (SASA) analysis assessed the proteins’ exposure to the solvent environment. The SASA values of T. castaneum (AChE) and S. litura (AChE) proteins (free and 3b-bound) were around 200–225 nm^2^ and 210–245 nm^2^, respectively, with slight fluctuations indicating sustained solvent exposure. The complex has a smaller and more stable SASA, showing that ligand binding reduces solvent exposure, either due to burial of hydrophobic areas or overall structural tightness (Fig. 10D). The number of hydrogen bonds, which indicates intra-protein interaction stability, was also investigated. The *T. castaneum* (AChE) and *S. litura* (AChE) proteins (free and **3b**-bound) systems remained within 135–165 and 130–170, respectively. AChE-3b complexes sustain a higher number of hydrogen bonds over time than the protein alone. This shows that **3b** binding creates more intermolecular hydrogen bonds, which may improve complex stability and specificity (Fig. 10E). To assess protein-ligand complex stability, intermolecular hydrogen bonds between the protein and the 3b ligand were investigated for 50 ns. All systems had 0–6 hydrogen bonds with modest variations, indicating that the protein-ligand interactions were stable (Fig. 10F). These findings are consistent with prior MD studies on anticancer protein-ligand systems, which also used RMSD, RMSF, SASA, and hydrogen bond metrics to assess complex stability and dynamic behavior^52–54^.

## Experimental

### Materials

Using a Gallenkamp apparatus, melting points (uncorrected) were determined in ^o^C. The infrared spectra (KBr) were obtained using a Thermo Scientific Nicolet iS10 FTIR spectrometer. Also, Bruker’s spectrometer 400 MHz (^1^H NMR), and 100 MHz (^13^C NMR) were used to estimate NMR spectra in DMSO-*d*_*6*_ as a solvent and an internal standard. At 70 eV, electron impact mass spectra were determined using the Varian MAT 311Kratos instrument.

### Chemical reactions

#### *The general procedure for the Preparation of* 3-(3-(benzofuran-2-yl)-1-phenyl-1*H*-pyrazol-4-yl)-2-cyano-*N*-(4-substitutedphenyl)-acrylamide

An alcoholic solution (20 ml) of compound **1** (2 gm, 0.01 mol) was refluxed with 2-cyano-*N*-(4-substituted phenyl)acetamide (**2a–f**) and (0.01 mol) for 2 h. in the presence of 0.5 ml of piperidine to afford crude compounds **3a–f**. The solid formed on hot was filtered off, scrubbed with hot EtOH, then purified from the EtOH: DMF (1:2) to give compounds **3a-f**.

#### 3-(3-(benzofuran-2-yl)-1-phenyl-1*H*-pyrazol-4-yl)-2-cyano-*N*-(4-tolyl)acrylamide (3a)

Pale yellow sheet, yield (85%), mp. 252 °C. IR spectrum, KBr, υ’, cm^− 1^: 3280 (N-H, stretch), 2217 (CN, stretch), 1645 (C = O, stretch), 1595 (C = N, stretch). ^1^H NMR spectrum (DMSO-*d*_6_), δ, ppm: 2.30 s (3 H, CH_3_), 7.20 (d, 2 H, Ar-H, *J* = 8 *Hz*), 7.35 (t, 1H, Ar-H), 7.43 (t, 1H, Ar-H, ), 7.51 (t, 1H, Ar-H), 7.56 (s, 1H, CH_furan_), 7.56 (d, 2 H, Ar-_H_, *J* = 8.4 *Hz*), 7.65 (t, 2 H, Ar-H, ), 7.72 (d, 1H, Ar-_H_, *J* = 8.2 *Hz*), 7.79 (d, 1H, Ar-_H_, *J* = 7.6 *Hz*), 7.98 (d, 2 H, Ar-_H_, *J* = 8 *Hz*), 8.59 s (1H, =CH), 9.26 s (s,1H, CH_pyrazole_), 10.37 (s,1H, NH). ^13^C NMR spectrum (DMSO -*d*_*6*_), δ_C_, ppm: 21.00, 106.78, 107.37, 111.99, 115.42, 117.13, 120.23 (2 C), 121.41, 122.35 (2 C), 124.16, 126.14, 128.32, 128.80, 129.60 (2 C), 129.99, 130.43 (2 C), 134.00, 136.17, 138.91, 141.36, 145.02, 148.27, 154.97, 160.39. MS (EI) *m/z* (*I*_*rel*_, %) for C_28_H_20_N_4_O_2_ (444.16): 444.79 (43.29) [*M*]^+^, 413.94 (100).

#### 3-(3-(benzofuran-2-yl)-1-phenyl-1*H*-pyrazol-4-yl)-2-cyano-*N*-(4-methoxyphenyl)acrylamide (3b)

Yellow sheet, yield (86%), mp. 250 °C. IR spectrum, KBr, υ’, cm^− 1^: 3339 (N-H, stretch), 2211 (CN, stretch), 1676 (C = O, stretch), 1594 (C = N, stretch). ^1^H NMR spectrum (DMSO-*d*_6_), δ, ppm: 3.77 s (3 H, OCH_3_), 6.97 (d, 2 H, Ar-H, *J* = 8.8 *Hz*), 7.35 (t, 1H, Ar-H, *J* = 7.6 *Hz*), 7.43 (t, 1H, Ar-H), 7.51 (t, 1H, Ar-H, ), 7.55 (s, 1H, CH_furan_), 7.61–7.67 (m, 4 H, Ar-H), 7.72 (d, 1H, Ar-_H_, *J* = 8 *Hz*), 7.79 (d, 1H, Ar-H, *J* = 7.6 *Hz*), 7.98 (d, 1H, Ar-H, *J* = 8 *Hz*), 7.98 (d, 2 H, Ar-H, *J* = 8 *Hz*), 8.59 s (1H, =CH), 9.25 s (s,1H, CH_pyrazole_), 10.31 (s,1H, NH). ^13^C NMR spectrum (DMSO -*d*_*6*_), δ_C_, ppm: 55.70, 106.77, 107.35, 112.00, 114.32, 115.45 (2 C), 117.14, 120.24 (2 C), 122.35, 123.09 (2 C), 124.16, 126.14, 128.33, 128.80, 129.99, 130.44 (2 C), 131.67, 138.93, 141.23, 145.01, 148.30, 154.98, 156.57, 160.20. MS (EI) *m/z* (*I*_*rel*_, %) for C_28_H_20_N_4_O_3_ (460.15): 460.40 (29.46) [*M*]^+^, 362.17 (100).

#### 3-(3-(benzofuran-2-yl)-1-phenyl-1*H*-pyrazol-4-yl)-*N*-(4-chlorophenyl)-2-cyanoacrylamide (3c)

Yellow sheet, yield (87%), mp. 278 °C. IR spectrum, KBr, υ’, cm^− 1^: 3275 (N-H, stretch), 2217 (CN, stretch), 1646 (C = O, stretch), 1592 (C = N, stretch). ^1^H NMR spectrum (DMSO-*d*_6_), δ, ppm: 7.35 (t, 1H, Ar-H, ), 7.40 (d, 1H, Ar-H, *J* = 7 *Hz*), 7.45 (t, 2 H, Ar-H), 7.51 (t, 1H, Ar-H), 7.57 (s, 1H, CH_furan_), 7.61 (t, 2 H, Ar-H), 7.72 (d, 1H, Ar-H), 7.76 (dd, 3 H, Ar-H, ), 7.98 (d, 2 H, Ar-H, *J* = 8 *Hz*), 8.62 s (1H, =CH), 9.27 s (s,1H, CH_pyrazole_), 10.57 (s,1H, NH). ^13^C NMR spectrum (DMSO -*d*_*6*_), δ_C_, ppm: 106.42, 107.44, 112.01, 115.36, 117.01, 120.25 (2 C), 122.35, 122.94 (2 C), 124.17, 126.16, 128.32, 128.57 (2 C), 128.83, 129.14, 130.09 (2 C), 130.43, 137.74, 138.89, 141.81, 145.08, 148.23, 154.98, 160.72. MS (EI) *m/z* (*I*_*rel*_, %) for C_27_H_17_ClN_4_O_2_ (464.10/ 466.10): 466.61 (29.25) [*M*]^+^+2, 270.94 (100).

#### 3-(3-(benzofuran-2-yl)-1-phenyl-1*H*-pyrazol-4-yl)-*N*-(4-bromophenyl)-2-cyanoacrylamide (3d)

Yellow sheet, yield (88%), mp. >280 °C. IR spectrum, KBr, υ’, cm^− 1^: 3275 (N-H, stretch), 2217 (CN, stretch), 1646 (C = O, stretch), 1596 (C = N, stretch). ^1^H NMR spectrum (DMSO-*d*_6_), δ, ppm: 7.35 (t, 1H, Ar-H, ), 7.43 (dd, 1H, Ar-H, *J* = 7.2 *Hz*), 7.51 (t, 1H, Ar-H), 7.56 (d, 2 H, Ar-H, *J* = 7 *Hz*), 7.59 (s, 1H, CH_furan_), 7.65 (t, 2 H, Ar-H, ), 7.69–7.73 (m, 3 H, Ar-H), 7.78 (d, 1H, Ar-H), 7.98 (d, 2 H, Ar-H, *J* = 8 *Hz*), 8.61 s (1H, =CH), 9.26 s (s,1H, CH_pyrazole_), 10.56 (s,1H, NH). ^13^C NMR spectrum (DMSO -*d*_*6*_), δ_C_, ppm: 106.45, 107.45, 112.01, 115.36, 116.71, 117.00 (2 C), 120.26, 123.29 (2 C), 124.17, 126.17, 128.32, 128.84, 130.11, 130.44 (3 C), 132.06 (2 C), 138.17, 138.90, 141.83, 145.09, 148.23, 154.98, 160.73. MS (EI) *m/z* (*I*_*rel*_, %) for C_27_H_17_BrN_4_O_2_ (508.05-510.05): 508.41/ 510.48 (12.73/ 15.64) [*M*]^+^ / [*M*]^+^+2, 468.28 (100).

#### 3-(3-(benzofuran-2-yl)-1-phenyl-1*H*-pyrazol-4-yl)-2-cyano-*N*-(4-nitrophenyl)acrylamide (3e)

Yellow powder, yield (84%), mp. 266 °C. IR spectrum, KBr, υ’, cm^− 1^: 3274 (N-H, stretch), 2217 (CN, stretch), 1646 (C = O, stretch), 1596 (C = N, stretch). ^1^H NMR spectrum (DMSO-*d*_6_), δ, ppm: 7.34 (t, 1H, Ar-_H_, *J* = 7.2 *Hz*), 7.42 (t, 1H, Ar-_H_, *J* = 7.6 *Hz*), 7.51 (t, 1H, Ar-_H_, *J* = 7.2 *Hz*), 7.57 (s, 1H, CH_furan_), 7.64 (t, 2 H, Ar-_H_, *J* = 7.6 *Hz*), 7.71 (d, 1H, Ar-_H_, *J* = 8 *Hz*), 7.77 (d, 1H, Ar-_H_, *J* = 8 *Hz*), 7.95–8.01 (m, 4 H, Ar-_H_), 8.29 (d, 2 H, Ar-_H_, *J* = 8.8 *Hz*), 8.66 s (1H, =CH), 9.25 s (s,1H, CH_pyrazole_), 10.96 (s,1H, NH). ^13^C NMR spectrum (DMSO -*d*_*6*_), δ_C_, ppm:106.00, 107.54, 111.91, 112.02, 115.27, 119.68 (2 C), 119.87 (2 C), 120.25, 120.90, 122.44 (2 C), 124.16, 126.88, 128.24, 130.42 (3 C), 138.84 (2 C), 142.57, 143.41, 145.21, 148.15, 158.23, 161.40. MS (EI) *m/z* (*I*_*rel*_, %) for C_27_H_17_N_5_O_4_ (475.13): 475.74 (9.55) [*M*]^+^, 267.13 (100).

#### 4-(3-(3-(benzofuran-2-yl)-1-phenyl-1*H*-pyrazol-4-yl)-2-cyanoacrylamido)benzoic acid (3f)

Yellow Sheet, yield (85%), mp. >280 °C. IR spectrum, KBr, υ’, cm^− 1^: 3333 (N-H, stretch), 2211 (CN, stretch), 1691 (COO, stretch), 1614 (C = O, stretch), 1598 (C = N, stretch). ^1^H NMR spectrum (DMSO-*d*_6_), δ, ppm: 7.35 (t, 1H, Ar-H) ,7.43 (dd, 1H, Ar-H), 7.51 (t, 1H, Ar-H, ), 7.58 (d, 2 H, Ar_− H_, *J* = 7.2 *Hz*), 7.60 (s, 1H, CH_furan_), 7.65 (t, 2 H, Ar-H, ), 7.70–7.73 (m, 3 H, Ar-H), 7.79 (d, 1H, Ar-H, *J* = 7.6 *Hz*), 7.98 (d, 2 H, Ar-H, *J* = 7.6 *Hz*), 8.62 s (1H, =CH), 9.27 s (s,1H, CH_pyrazole_), 10.56 (s,2 H, NH, OH). ^13^C NMR spectrum (DMSO -*d*_*6*_), δ_C_, ppm: 106.45, 107.45, 112.02, 115.36, 116.70, 117.00 (2 C), 120.26, 122.35 (2 C), 123.28, 124.17, 126.16, 128.33, 128.83, 130.11, 130.44 (2 C), 132.06 (2 C), 138.18, 138.90, 141.83, 145.09, 148.23, 154.99, 160.73 (2 C). MS (EI) *m/z* (*I*_*rel*_, %) for C_28_H_18_N_4_O_4_ (474.13): 474.31 (17.13) [*M*]^+^, 319.22 (100).

#### 3-(3-(benzofuran-2-yl)-1-phenyl-1*H*-pyrazol-4-yl)-2-cyano-*N*-(4-phenylthiazol-2-yl)acrylamide (5)

Orange powder, yield (81%), mp. 145 °C. IR spectrum, KBr, υ’, cm^− 1^: 3403 (OH, stretch), 2207 (CN, stretch), 1676 (C = O, stretch), 1583 (C = N, stretch). ^1^H NMR spectrum (DMSO-*d*_6_), δ, ppm: 7.37–7.47 (d, 2 H, Ar-H, *J* = 7 *Hz*), 7.52–7.56 (m, 4 H, Ar-H, ), 7.73–7.76 (d, 2 H, H_fura_, *J* = 7.6 *Hz* ) ,7.78–7.81 (d, 2 H, Ar-H, *J* = 7.4 *Hz)*, 7.83–7.95 (m, 4 H, Ar-H), 7.98–7.99 (m, 2 H, Ar-H, H_thiazole_), 8.75 s (1H, =CH), 9.29 s (s,1H, CH_pyrazole_), 13.16 (s,1H, OH). MS (EI) *m/z* (*I*_*rel*_, %) for C_30_H_19_N_5_O_2_S (513.13): 513.56 (30.87) [*M*]^+^, 329.83 (100).

### Insecticidal bioassays

#### Bioassay of *Spodoptera littoralis* and *Tribolium castaneum*

Contact toxicity assays were employed as the method of application in this study. Each compound was dissolved in acetone to prepare serial dilutions. For both insect species, concentrations of 15.62, 31.25, 62.5, 125, 250, 500, and 1000 ppm of each compound were tested. The chemicals were applied to Petri dishes (9 cm in diameter) using 1.5 mL per dish, with the dish gently rotated in circles to ensure even distribution. Control dishes were treated with 1.5 ml of acetone alone. The acetone was allowed to evaporate for 30 min, leaving a thin film of the compound on the dish surface prior to introducing the insects. For the bioassays, five unsexed adults of *S. littoralis* (4th instar larvae) and *T. castaneum* (adults) were placed into the dishes, either separately or together. Controls were maintained under identical conditions without the compounds. Each treatment was replicated three times. Mortality was recorded at 24, 48, and 72 h post-treatment and corrected using Abbott’s formula. Insects were considered dead when no movement was observed. The LC_50_ values were determined through probit analysis^55,56^.

#### Statistical analysis

Data were presented as mean ± standard error (SE). Corrected mortality rates were analyzed using probit analysis to estimate LC_5_, LC_50_, and LC_95_ values, along with their fiducial limits, employing the Finney method in LCP-line software. The goodness of fit was evaluated using the chi-square test^57^.

### Molecular docking analysis

#### Molecular docking of synthesized compounds

To determine the compounds’ biological potential, their binding interactions with specific protein targets were evaluated using molecular docking (Table 11). The three-dimensional structures of the *T. castaneum* (AChE) and *S. litura* (AChE) proteins were modelled using Modeller v.10.8 and preprocessed with PyMOL. Hydrogen atoms were added, and the structures created were stored in pdbqt format with Auto Dock Vina. The ligand structures were energy reduced and translated to mol2 format using Open Babel. Docking simulations were run with Auto Dock Vina using ligand-centered maps prepared by the Auto Grid software. Following docking, the resulting protein-ligand complexes were studied. Discovery Studio 4.5 was used to generate two-dimensional interaction diagrams, and ADMETlab 3.0 was used to estimate the compounds’ physicochemical and ADMET attributes^58,59^.

**Table 11:** List of target proteins, PDB IDs, active site coordinates, and references.

Protein targets	Accesion number	Active site coordinates:			Reference standared	Co-crystallized inhibitor	RMSD values (Å)	Common residues
		X	Y	Z				
*T. castaneum* (AChE)	NP_001280534.1	13.10	5.22	− 0.56	Carbaryl	(NAF)	1.02	Val360 Ser213
*S. litura* (AChE)	NP_022825018.1	2.19	− 14.88	6.28	Carbaryl	(NAF)	1.30	Val313 Phe9

#### Molecular dynamics (MD) simulation

Molecular dynamics (MD) simulations were conducted to assess the stability and interaction dynamics of protein-ligand complexes using GROMACS 2018 with the CHARMM36 force field for protein topology and the CGenFF server (Geoff) for ligand parameters. During equilibration, ligand positions were constrained in NVT (constant particle number, volume, and temperature) and NPT (constant particle number, pressure, and temperature) ensembles, each run for 1000 ps at 300 K and 1.0 bar to ensure system stability. Post-simulation analyses, including Root Mean Square Deviation (RMSD), Root Mean Square Fluctuation (RMSF), and radius of gyration (Rg), were used to evaluate structural stability, residue-level flexibility, and overall compactness of the complexes^60^.

## Conclusions

The current effort aims to synthesize and evaluate the molecular modeling and insecticide activity of novel benzofuran-pyrazole derivatives in order to uncover new structural leads acting as agents. Highly poisonous compounds **3a**,** 3b**, and **3c** showed the most decisive action against the insecticidal activity towards *Spodoptera littoralis* and *Tribolium castaneum* according to the biological experiment data. The LC_50_ values were 52.94, 36.11, 49.57, and 55.47 ppm at 72 h for compounds **1**,** 3a**,** 3b**, and **3c**, respectively, while compounds **3d**,** 3e**,** 3f**, and **5** were not lethal to *S. littoralis*. LC_50_ values of 79.58, 106.37, and 226.17 ppm at 72 h for compounds **3b**, **3c**, and **3a**, respectively, while in contrast, compounds **1**, **3d**,** 3e**,** 3f**, and **5** exhibited very low or no contact toxicity against *T. castaneum*. DFT calculations confirmed that stereochemistry plays a decisive role in the electronic properties of the synthesized compounds. The E forms of **3a**, **3c**, **3d**, and **3e** exhibited lower energy gaps and higher softness and electrophilicity, with **3c**.E was identified as the most electronically reactive species, while **3b**, **3f**, and **5** favored the Z form with enhanced orbital overlap and dipole moments. Although **3c**.E showed superior electronic reactivity, molecular docking highlighted **3b** as the strongest AChE binder due to more favorable steric fitting and interaction patterns within the active site. Accordingly, the combined results confirm that DFT reactivity and docking affinity reflect different aspects of molecular performance, with **3c.E** standing out electronically and **3b** emerging as the most promising target-specific binder. In addition, compounds **3a–c** interact with key catalytic and hydrophobic residues in both *S. litura* and *T. castaneum* AChE. While in silico ADMET profiles suggest favorable drug-like properties and low toxicity risks, high lipophilicity may affect solubility. These results strongly support further experimental validation of these compounds as novel insecticide candidates.

## Supplementary Information

Below is the link to the electronic supplementary material.

Supplementary Material 1

## Data Availability

Data will be made available on request from Corresponding author.
