# Supplementary material for: Design, characterization, DFT studies, and molecular docking of new benzofuran–pyrazol-acrylamide hybrids as insecticidal agents against Spodoptera littoralis and Tribolium castaneum
Source: Sci Rep. 2026 Mar 25;16:10344. doi: 10.1038/s41598-026-39839-z (PMC13031331; doi:10.1038/s41598-026-39839-z)
Supplement: Supplementary file 1 — Supplementary Material 1 [file 41598_2026_39839_MOESM1_ESM.docx]

**Design, Characterization, DFT studies, and Molecular docking of New Benzofuran–pyrazol-acrylamide Hybrids as Insecticidal agents against *Spodoptera littoralis* and *Tribolium castaneum***

**Ghada G. El-Bana^a^*^, b^, Mohamed R. Fouad^c^,** **Ahmed D.H. Deeb^d^, Ahmed M. Wahba^e,f^, Ahmed F. El-Sayed^g,h^, Ghada E. Abd El Ghani^a^**

^a^Department of Chemistry, Faculty of Science, Mansoura University, El-Gomhoria Street, Mansoura ET- 35516, Egypt.

^b^Mansoura University Student's Hospital, Mansoura University, El-Gomhoria Street, Mansoura ET- 35516, Egypt.

^c^Department of Pesticide Chemistry and Technology, Faculty of Agriculture, Alexandria University, Aflaton St., 21545, El-Shatby, Alexandria, Egypt

^d^Department of Physical Sciences, Chemistry Division, College of Science, Jazan University, Jazan 45142, Saudi Arabia.

^e^Medical Sciences & Preparatory Year Department, North Private College of Nursing, Arar 73312, Northern Border Province, Kingdom of Saudi Arabia

^f^Basic Science Department, Higher Institute of Engineering and Technology (HIET), El-Mahala EL-Kobra 31951, Egypt.

^g^Microbial Genetics Department, Biotechnology Research Institute, National Research Centre, Giza, Egypt;

^h^Microbial Genetics, Egypt Center for Research and Regenerative Medicine (ECRRM), Cairo, Egypt

*Corresponding author Email: [ghadaelbana@mans.edu.eg](mailto:ghadaelbana@mans.edu.eg)


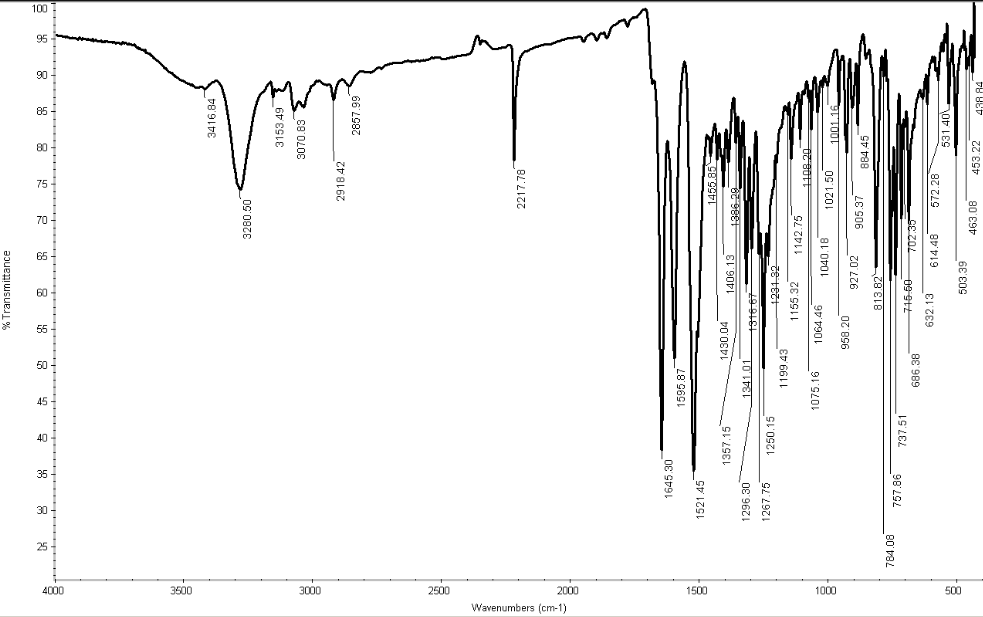

**Figure S1: IR spectrum of compound 3a**


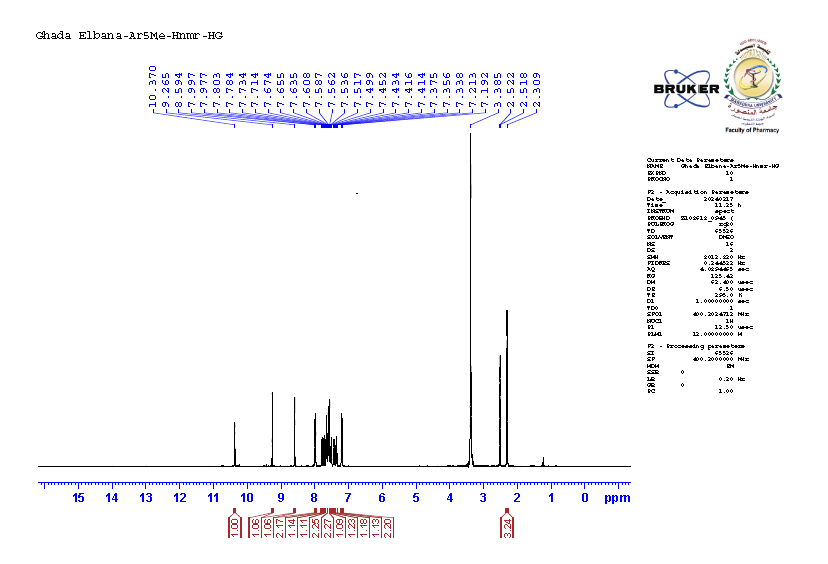

**Figure S2: ^1^H NMR spectrum of compound 3a**


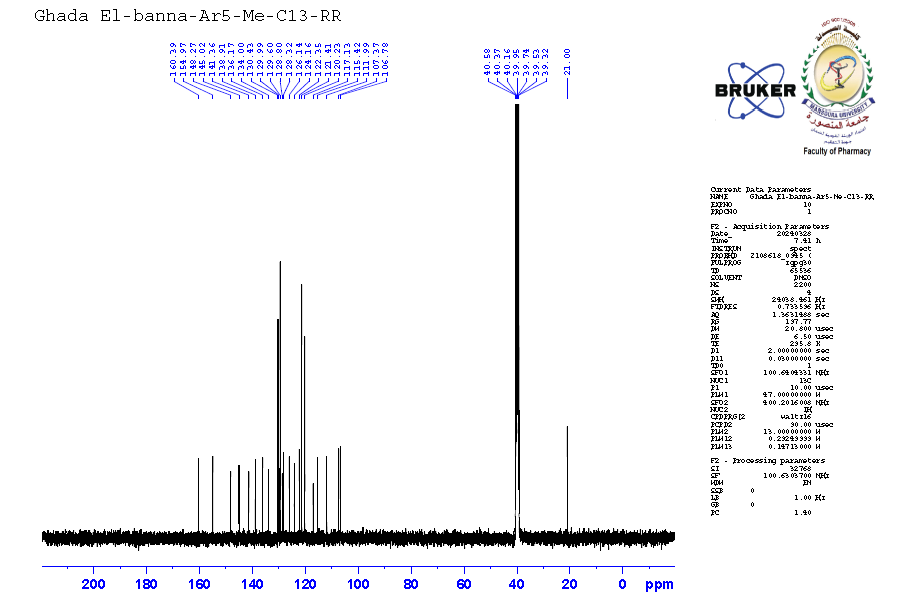

**Figure S3: ^13^C NMR spectrum of compound 3a**

**Figure S4: Mass spectrum of compound 3a**


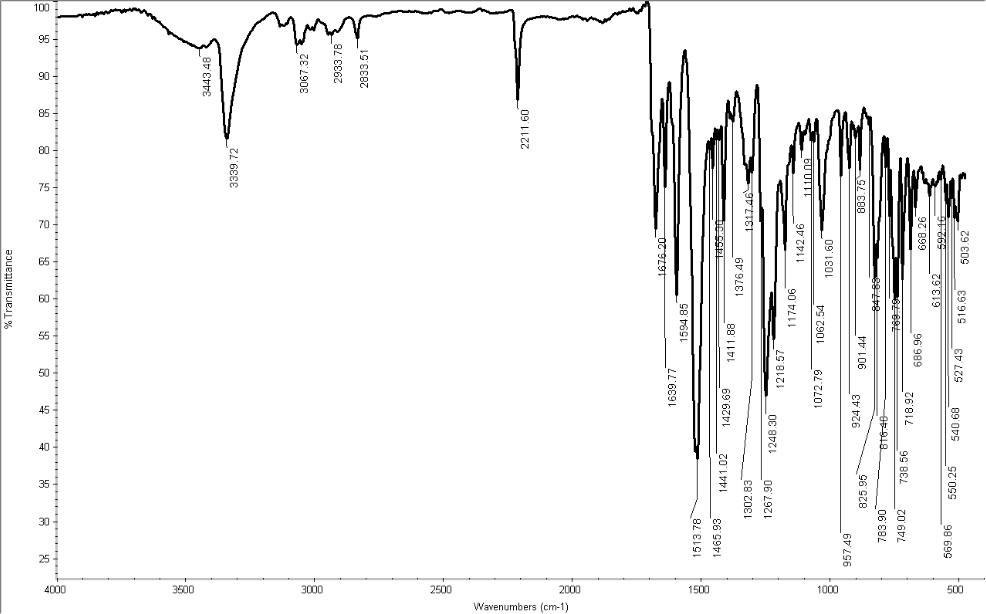

**Figure S5: IR spectrum of compound 3b**


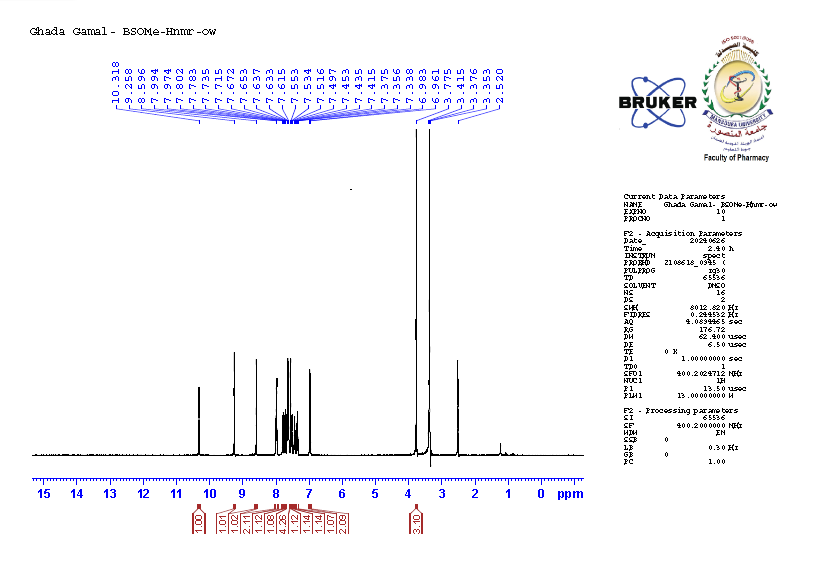

**Figure S6: ^1^H NMR spectrum of compound 3b**


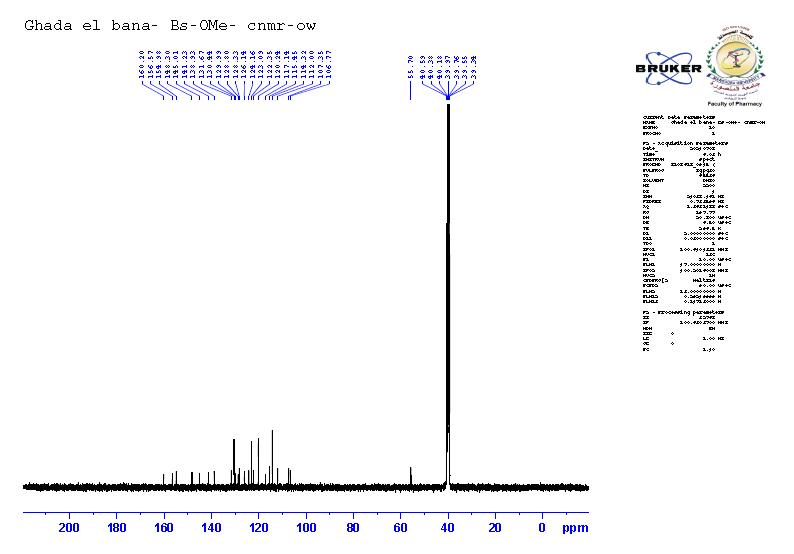

**Figure S7: ^13^C NMR spectrum of compound 3b**

**Figure S8: Mass spectrum of compound 3b**


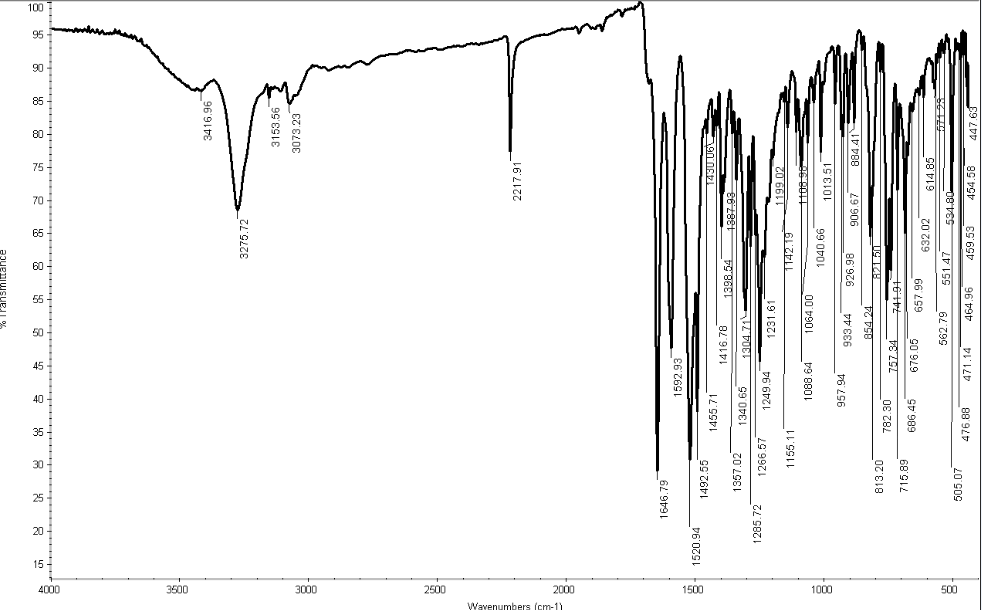

**Figure S9: IR spectrum of compound 3c**

**
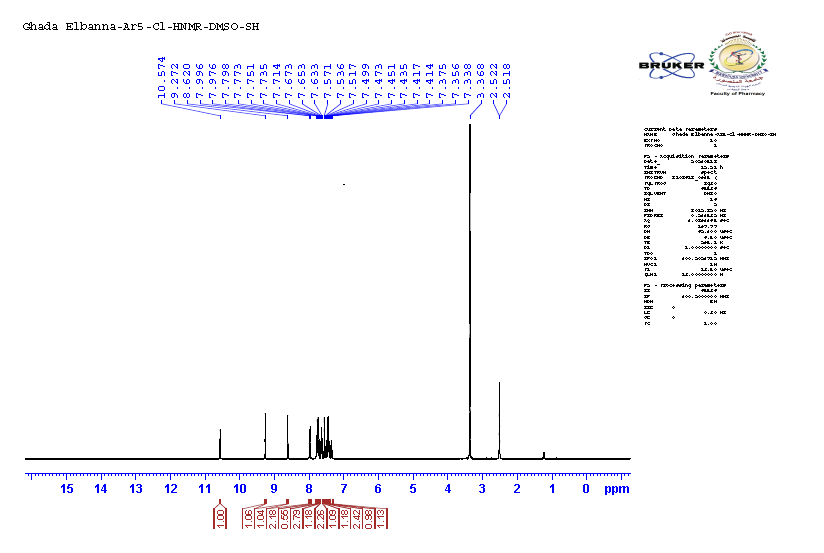
**

**Figure S10: ^1^H NMR spectrum of compound 3c**

**
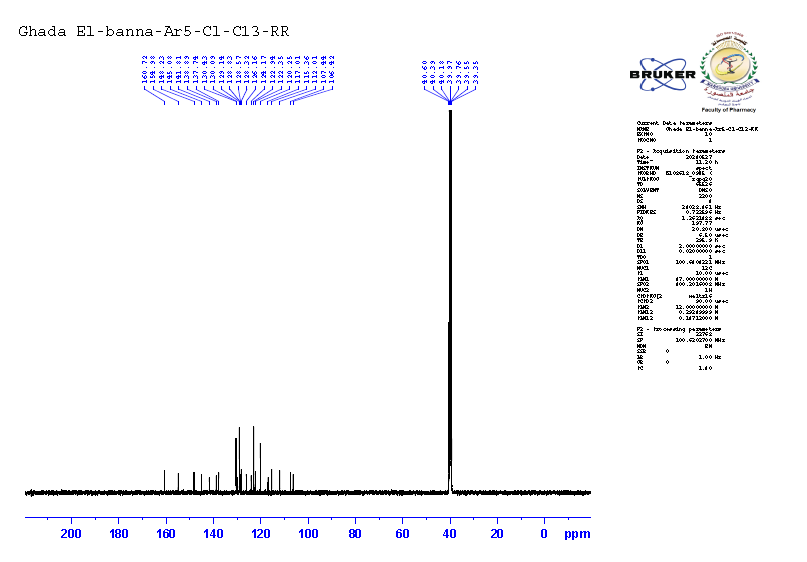
**

**Figure S11: ^13^C NMR spectrum of compound 3c**

**Figure S12: Mass spectrum of compound 3c**


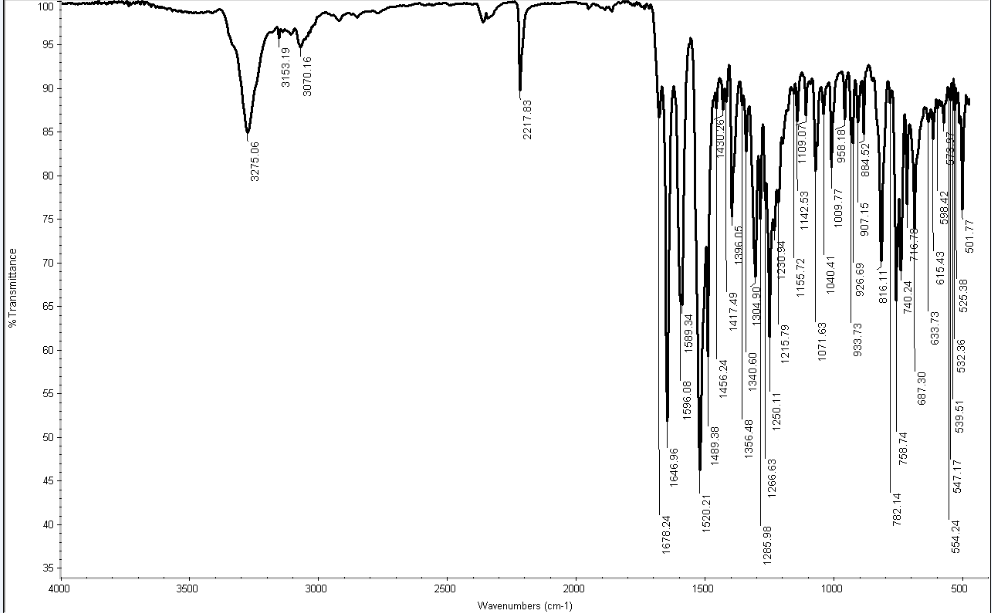

**Figure S13: IR spectrum of compound 3e**

**
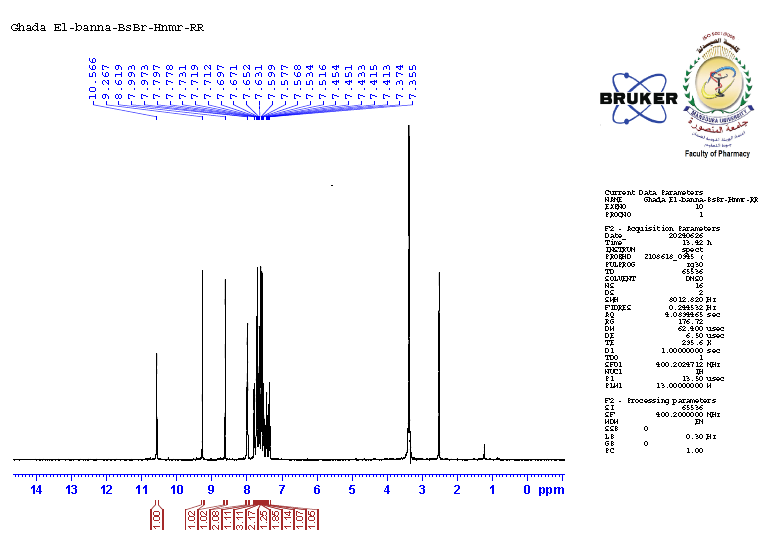
**

**Figure S14: ^1^H NMR spectrum of compound 3d**

**
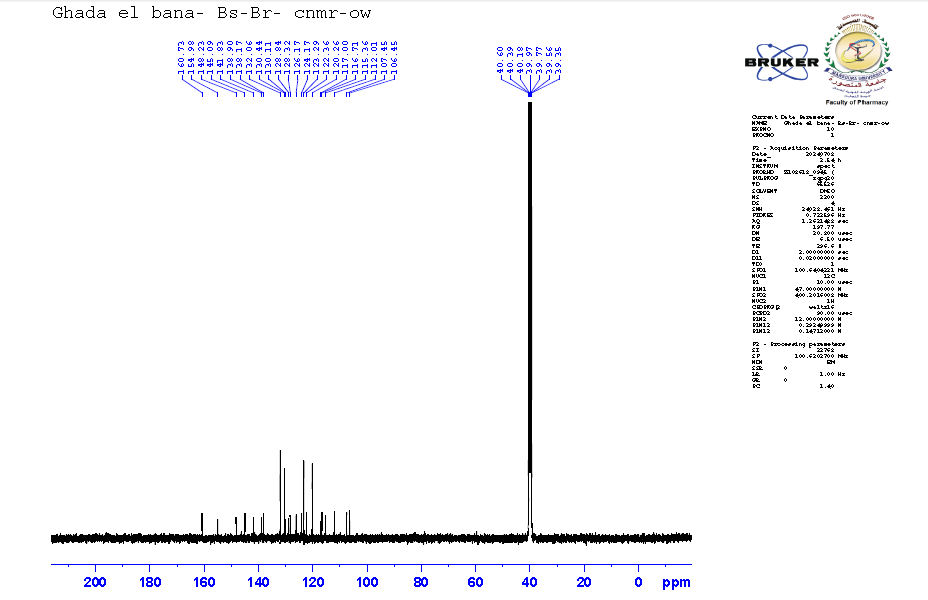
**

**Figure S15: ^13^C NMR spectrum of compound 3d**

**Figure S16: Mass spectrum of compound 3d**


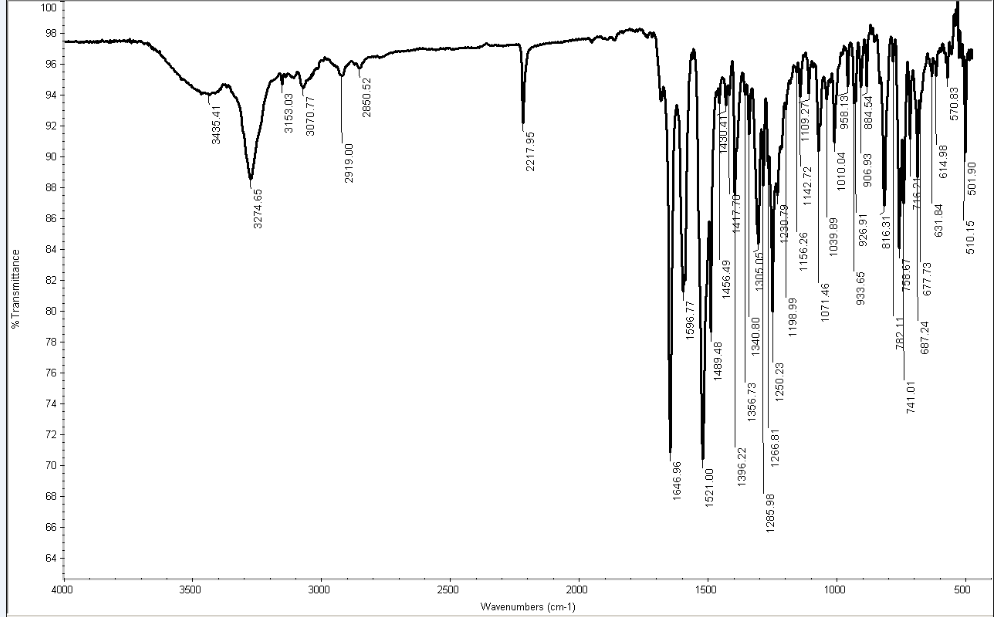

**Figure S17: IR spectrum of compound 3e**

**
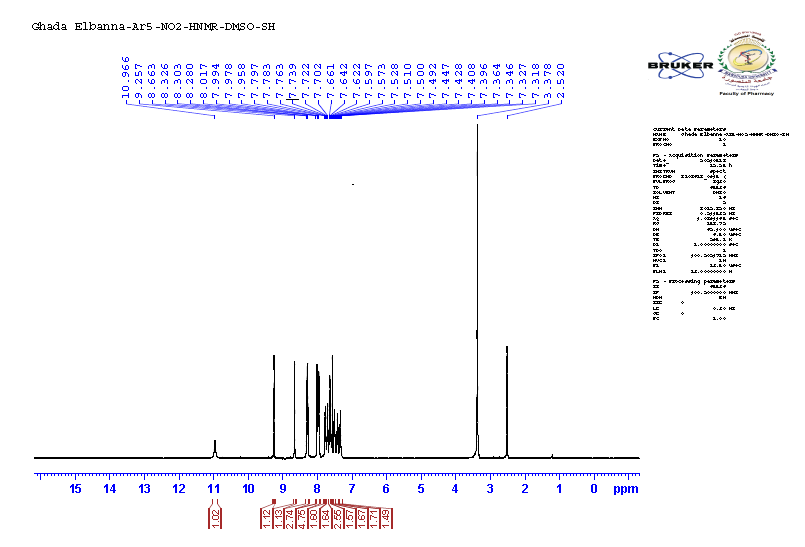
**

**Figure S18: ^1^H NMR spectrum of compound 3e**

**
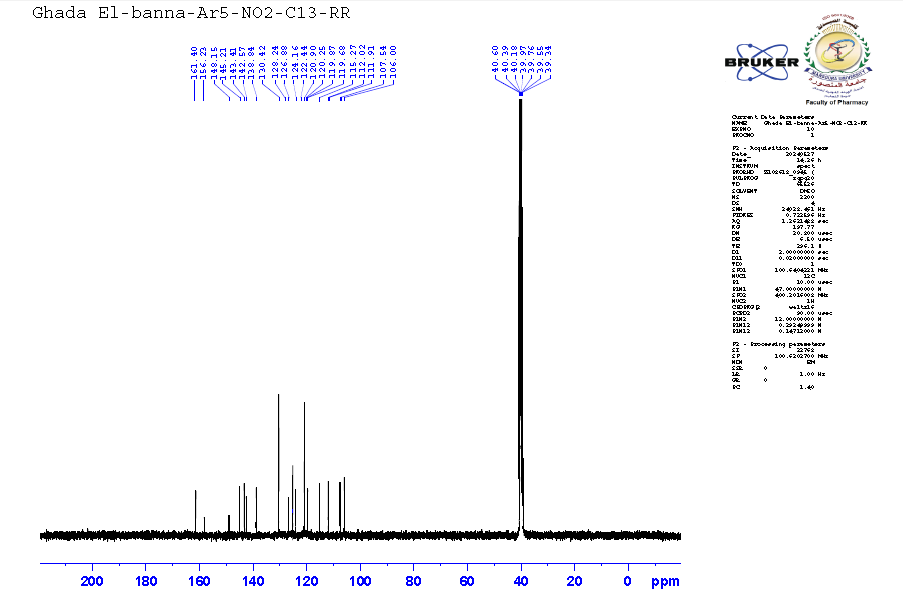
**

**Figure S19: ^13^C NMR spectrum of compound 3e**

**Figure S20: Mass spectrum of compound 3e**


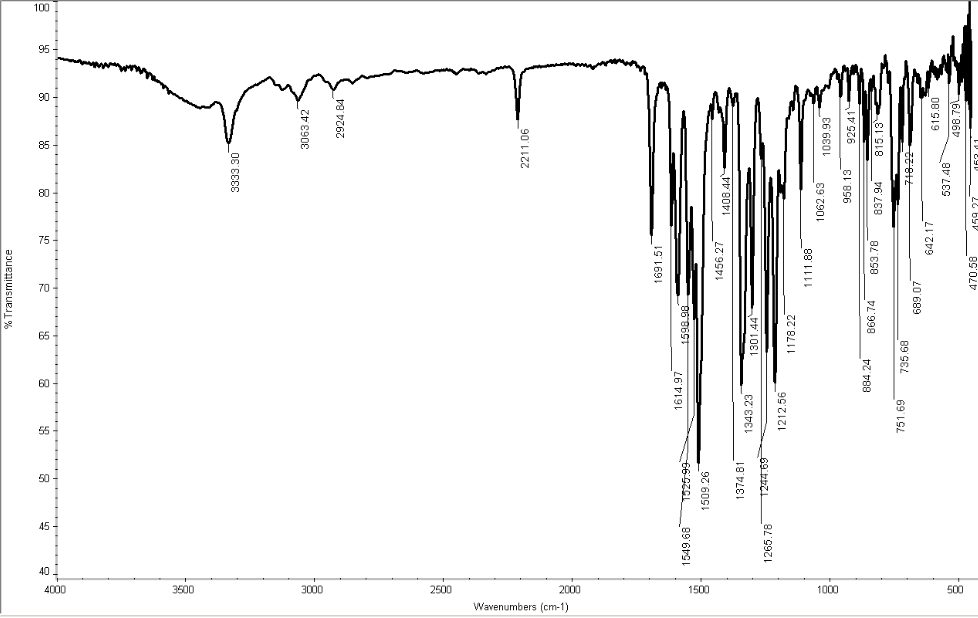

**Figure S21: IR spectrum of compound 3f**

**
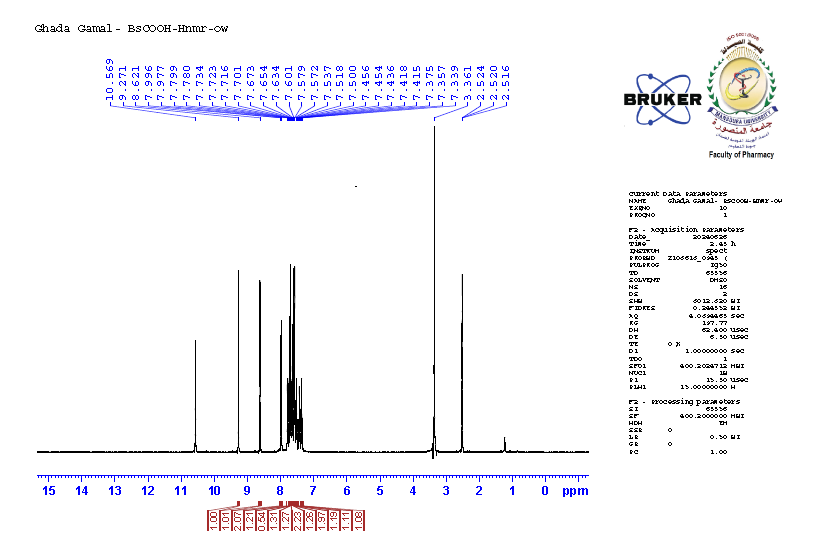
**

**Figure S22: ^1^H NMR spectrum of compound 3f**

**
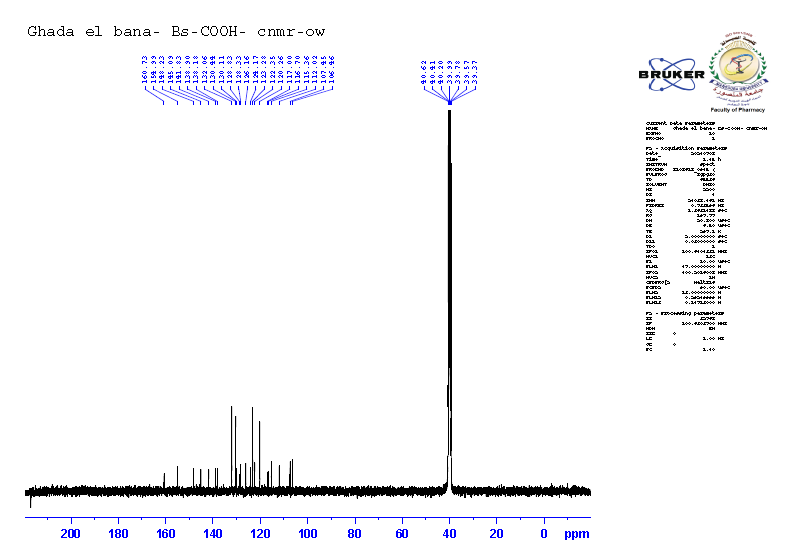
**

**Figure S23: ^13^C NMR spectrum of compound 3f**

**Figure S24: Mass spectrum of compound 3f**


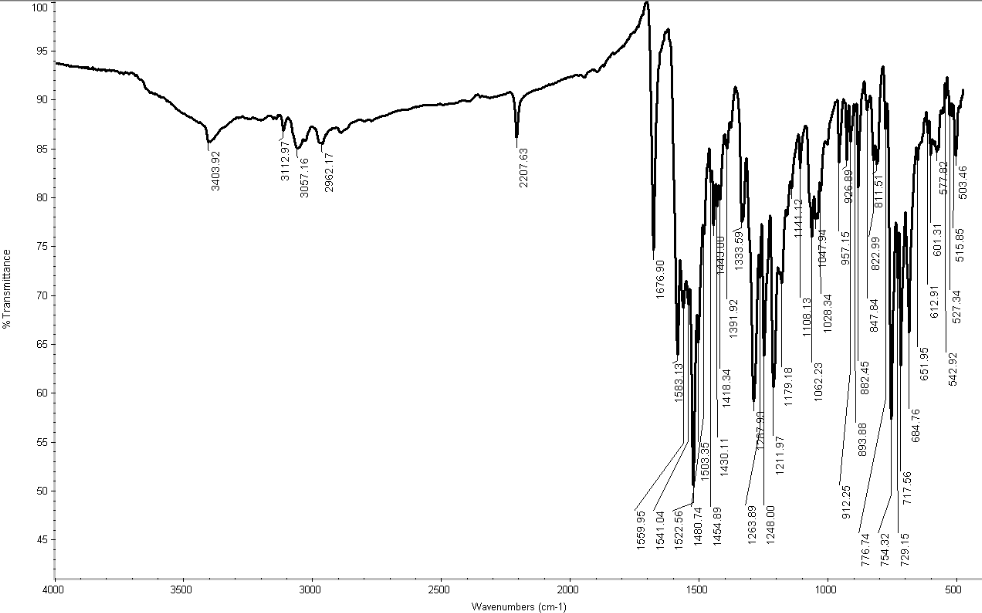

**Figure S25: IR spectrum of compound 5**


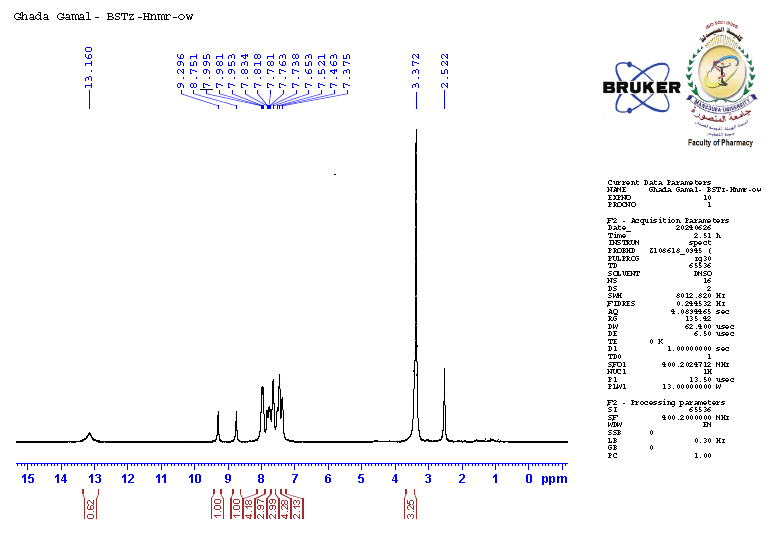

**Figure S26: ^1^H NMR spectrum of compound 5**

**Figure S27: Mass spectrum of compound 5**
